# Chemically Interpretable Explanations for Molecular Property Prediction via Fragment-Level Shapley Values

**DOI:** 10.1021/acs.jcim.6c01425

**Published:** 2026-08-14

**Authors:** Jannik P. Roth

**Affiliations:** † Mulliken Center for Theoretical Chemistry, University of Bonn, Bonn 53115, Germany; ‡ Life and Medical Sciences (LIMES) Institute, University of Bonn, Bonn 53115, Germany; § Bonn Center for Mathematical Life Sciences, University of Bonn, Bonn 53115, Germany

## Abstract

Machine learning has emerged as a powerful approach for molecular property prediction and drug discovery. However, the black-box nature of many machine learning models limits their interpretability, trustworthiness, and adoption in interdisciplinary research settings. This is especially the case in molecular machine learning, where explanations of model predictions are used for informed decision-making and downstream tasks. Shapley values, originating from cooperative game theory, provide a principled framework for attributing model predictions to individual input features. However, existing Shapley value-based explanations for molecular machine learning often rely on sampling-based approximations or operate at the level of abstract features, which can reduce attribution stability and limit chemical interpretability and actionability. Here, we introduce a fragment-level Shapley value framework that enables the exact computation of feature contributions at the level of chemically meaningful fragments for molecular property predictions without relying on sampling or feature imputation. By decomposing molecules into fragments, the proposed approach yields actionable explanations that can be directly related to established chemical concepts. We apply the method post hoc to random forest and graph convolutional network models using common molecular representations, including extended-connectivity fingerprints and molecular graphs. The approach is evaluated across three representative property prediction tasks: aqueous solubility, mutagenicity, and antiviral potency. Fragment-level Shapley values reproduce well-established chemical trends, identify known toxicophores, and enable guided molecular optimization. In addition, the method provides insights into model learning characteristics and helps delineate the applicability domain, particularly in settings with limited and structurally biased data. Overall, this work demonstrates that adapting Shapley values to chemically meaningful fragments enables interpretable explanations for molecular machine learning models, supporting molecular optimization and model validation.

## Graphical abstract

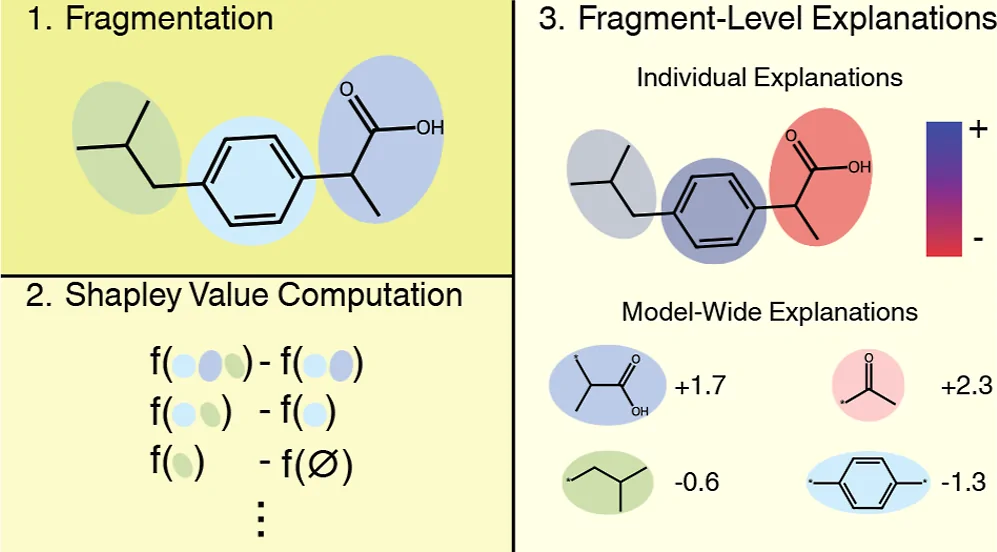

## 1 Introduction

Machine learning (ML) models have been adopted across numerous scientific disciplines, including chemistry and pharmaceutical sciences,^1–5^ where they are widely used for molecular property prediction and drug development. In particular, the use of deep learning has enabled new applications in the pharmaceutical sciences,^3,5,6^ such as enhancing drug discovery^7^ and accelerating molecular design through data-driven approaches.^8,9^ Despite their predictive power, many high-performing ML models operate as black boxes, making their predictions difficult to rationalize from a human perspective. While simpler and more interpretable models exist, they often do not achieve comparable predictive performance on complex molecular tasks, motivating the use of more expressive but less interpretable approaches. This limited interpretability complicates adoption for nonexperts, restricts their use in interdisciplinary research settings, and reduces the ability to assess whether a model will generalize reliably to new data.^10,11^ Consequently, explainable artificial intelligence (XAI) has emerged as an active research area focused on developing methods to rationalize model predictions.^12–14^ Among the many XAI approaches that have been developed,^15–17^ feature-attribution methods have become a widely used class of explanation techniques, as they provide a direct mapping between input features and model outputs and can be applied to a broad range of models. These methods aim to assign an importance score to each input feature with respect to the model’s prediction, thereby identifying features that most strongly influence the model’s decision. Prominent examples include SHapley Additive exPlanations (SHAP),^18,19^ which approximate Shapley values for tabular models, and GNNExplainer,^20^ which identifies important substructures in graph neural networks. While these methods have proven useful, they typically operate at the level of abstract features or provide unsigned importance scores, which can limit their interpretability and chemical actionability.

One of the most widely used feature-attribution approaches is based on the Shapley value framework.^21^ Originating from cooperative game theory, Shapley values provide a principled method for fairly attributing the outcome of a collaborative game to its players. When adapted to ML, they assign to each input feature its contribution to a model prediction, offering a theoretically founded and model-agnostic approach to interpretation. Their strong theoretical properties have contributed to their widespread adoption across many research disciplines, including drug discovery and cheminformatics.^14,22,23^ Applying Shapley values to high-dimensional molecular representations introduces several challenges. First, the exact computation of Shapley values scales factorially with the number of input features and is therefore often infeasible in molecular ML, where feature spaces can comprise hundreds to thousands of dimensions. As a result, Shapley values are typically approximated using sampling-based approaches^24,25^ or model-specific optimizations.^18,19,22^ Although methods for exact Shapley value computation have been developed, these approaches are generally restricted to specific model classes or feature representations.^26–28^ Second, the Shapley value formalism requires evaluating the model on arbitrary subsets of input features. However, most ML models are defined only for complete input representations and cannot be directly evaluated on such partial inputs. This limitation is typically addressed using feature imputation strategies,^29^ which introduce additional assumptions and may affect attribution reliability.

Furthermore, feature-level attributions derived from common molecular representations, such as fingerprints or graphs, do not necessarily correspond to chemically meaningful substructures, limiting their interpretability and making it difficult to translate them into concrete molecular design strategies. To address these limitations, several XAI approaches have been adapted to molecular ML, including counterfactual explanations,^30–32^ contrastive explanations,^33^ and anchors.^34,35^ These methods combine XAI techniques with fragment-based representations, enabling explanations at the level of chemically meaningful substructures and improving their interpretability and applicability in downstream tasks.^36^ Related fragment-based attribution approaches have also been proposed.^37–39^ Among these, MolSHAP represents the conceptually closest prior work, as both approaches aim to obtain Shapley-based explanations at the level of chemically meaningful molecular substructures.^37^ However, MolSHAP estimates R-group contributions within predefined analogue series and is therefore particularly suited for structure–activity relationship (SAR) analysis of congeneric compounds. In contrast, the present approach does not require analogue-series definitions, scaffold identification, or scaffold–R-group decompositions, but explains individual molecular predictions by treating molecular fragments directly as players in the Shapley value formalism. Other fragment-wise explainability methods often rely on specialized graph neural network architectures, masking procedures, or substructure pooling strategies, which can limit their applicability to already trained models or to nongraph molecular representations.^38,39^ Similarly, recent work combining attribution methods with uncertainty quantification^40^ focuses on comparing posthoc attribution techniques for graph neural network predictions and predictive uncertainty, whereas the present work reformulates the Shapley game itself at the level of chemically meaningful fragments. The key methodological novelty of the proposed framework is the exact, posthoc computation of fragment-level Shapley values for trained models operating on common molecular representations, including fingerprints and molecular graphs.

In this work, we develop a posthoc method for computing fragment-level Shapley values for machine learning models operating on common molecular representations, including extended-connectivity fingerprints and molecular graphs. By leveraging molecular fragmentation, the number of players in the Shapley value formalism is reduced to a tractable size, typically fewer than ten fragments for drug-like molecules, enabling exact Shapley value computation without relying on approximation techniques. The proposed method is model-agnostic within these representation classes and can be applied post hoc to already trained models without requiring architectural modifications or retraining. We demonstrate that this framework yields chemically meaningful and actionable explanations across several representative molecular property prediction tasks. In particular, fragment-level Shapley values enable validation of learned structure–property relationships against established chemical knowledge, support model diagnostics such as identifying dominant structural motifs and applicability domains, and facilitate rational molecular design through fragment-level modifications.

## 2 Methods

### 2.1 Molecular Representations

In this work, two commonly used molecular representations are employed: extended-connectivity fingerprints (ECFPs)^41^ and molecular graphs.^42^ ECFPs encode the presence of local atom environments by hashing each unique environment to an integer value. Folded ECFPs generate a fixed-length representation by applying a modulo operation to these integer values and setting the corresponding positions in a binary vector to one, while all remaining entries are set to zero. This representation captures the presence of local atom environments and allows the identification of corresponding substructures in the molecular structure. ECFPs can be used with any ML algorithm that operates on tabular data. Throughout this study, ECFPs with a length of 2048 and a radius of 2 were used, although the presented interpretation framework is independent of these parameters. We employ molecular graphs as input representations for graph neural networks. In this work, each node corresponds to an atom and is characterized by the following features: atomic number, chirality, degree (number of directly bonded neighbors), formal charge, number of hydrogen atoms, number of radical electrons, hybridization, aromaticity, and ring membership. Edges in the graph correspond to chemical bonds in the molecule.

### 2.2 Shapley Values for Model Explanations

The Shapley value formalism originating from cooperative game theory can be adapted for ML models as follows: players correspond to input features (or, in the fragment-based formulation introduced below, molecular fragments) and the game corresponds to the prediction task of a model. The Shapley value of a feature quantifies its average marginal contribution across all possible coalitions of features and is defined as

1 $$\phi_{i}\left(\nu \right)=\sum_{S\subseteq F\backslash \left\{i\right\}}\frac{\left|S\right|!\left(\left|F\right|-\left|S\right|-1\right)!}{\left|F\right|!}\left(\nu \left(S\cup \left\{i\right\}\right)-\nu \left(S\right)\right)$$

where F denotes the complete feature set, S a coalition of features, ν(S) the value function defining the model output for the feature set S , and ϕ_*i*_(ν) the Shapley value of feature *i*. A key property of Shapley values is the efficiency property, which ensures that the sum of all Shapley values equals the model prediction relative to a defined baseline.

### 2.3 Exact Shapley Values via Molecular Fragmentation

We propose a fragment-level formulation of the Shapley value framework that enables exact attribution for molecular machine learning models. In contrast to conventional feature-based formulations, where players correspond to individual input features (e.g., fingerprint bits or graph nodes), we redefine players as chemically meaningful molecular fragments. This reinterpretation aligns the Shapley value formalism with chemical structure and allows attribution at the level of functional substructures rather than abstract features. Drug-like molecules can typically be decomposed into a small number of synthetically meaningful fragments that serve as interpretable and chemically intuitive contributors to the model prediction. Importantly, this reduces the number of players in the Shapley value formalism, often to fewer than ten fragments per molecule, rendering exact computation tractable. Molecular fragmentation was performed using the BRICS algorithm^43^ as implemented in RDKit,^44^ which generates fragments based on retrosynthetically motivated rules. The proposed approach is not restricted to a specific fragmentation scheme and can, in principle, be applied using alternative fragmentation methods.^45,46^ Applying the Shapley value formalism to molecular fragments yields exact Shapley values for each fragment with respect to the model prediction.

A key requirement of the Shapley value framework is the evaluation of the model on arbitrary subsets of players. In molecular settings, this corresponds to representing partial molecules composed of selected fragments. The construction of such fragment subsets depends on the underlying molecular representation. For ECFP-based models, fingerprints are first generated for the intact molecule. Each fingerprint bit is then associated with the central atom and corresponding atom environment from which it originated and assigned to the fragment containing the corresponding central atom. Importantly, atom environments are not recomputed on isolated fragments. Consequently, atom environments centered near BRICS cleavage sites may extend across fragment boundaries and are retained as part of the fragment containing the central atom. For a given fragment subset, all fingerprint bits associated with atoms belonging to the selected fragments are retained, while all remaining bits are set to zero. Under this construction, the union of all fragment-associated fingerprint bits exactly recovers the original molecular fingerprint (up to the standard bit-collision effects inherent to folded fingerprints). An all-zero fingerprint thus corresponds to an empty fragment set. For graph-based representations, fragment coalitions are constructed after featurization of the intact molecular graph. Atoms belonging to the selected fragments are retained as nodes, edges between retained atoms preserve the feature vectors assigned in the intact molecular graph, and all other nodes and incident edges are removed. Node and edge features are not recomputed after fragment removal. Thus, retained atoms preserve the features assigned in the intact molecule, including degree, hydrogen count, formal charge, and hybridization. This choice avoids generating artificial rehydrogenated or resanitized fragment structures, but it also means that partial graph inputs may contain boundary atoms whose feature vectors reflect the original molecular context. In addition, coalitions containing nonadjacent fragments can yield disconnected graphs. These graph coalitions should therefore be interpreted as model inputs representing subsets of the original molecular graph rather than as chemically valid standalone molecules. In many existing Shapley-based approaches, missing features are handled via feature imputation or sampling-based approximations to estimate contributions. In contrast, the proposed fragment-based representation enables direct model evaluation on well-defined fragment subsets, allowing exact Shapley values to be computed without relying on such approximations.

Exact fragment-level Shapley values are computed using eq 1 by exhaustively evaluating the model on all possible subsets of fragments. Here, each subset corresponds to a coalition of fragments included in the prediction. For regression tasks, the model output is used directly as the value function ν. For classification tasks, the model output is transformed into logit (log-odds) space prior to Shapley value computation, ensuring the efficiency property. The value of the empty coalition, ν­(Ø), is defined as the model prediction for an input in which no fragments are present. For ECFP-based models, this corresponds to an all-zero fingerprint indicating the absence of all atom environments. For graph-based models, an empty input is approximated by a minimal graph consisting of a single node with all features initialized to zero and a self-loop edge, ensuring compatibility with the model architecture. This construction differs from a valid single-atom fragment, as it does not correspond to any chemical element or meaningful molecular structure. While such an “empty” molecule has no physical interpretation, it provides a well-defined reference point for attributing fragment-level contributions. This choice avoids the need for reference inputs derived from data set statistics (e.g., mean or background samples), which are commonly used in approximate Shapley methods to handle missing features. The resulting reference prediction, ν­(Ø), depends on the trained model and the chosen representation of the empty coalition. Consequently, absolute attribution magnitudes should be interpreted relative to this baseline, whereas relative fragment rankings and qualitative trends are generally preserved under reasonable changes to the baseline definition. In practice, alternative baseline definitions can be adopted depending on the intended interpretation. Their influence on the resulting fragment-level attributions is examined in a sensitivity analysis provided in the Supporting Information.

### 2.4 Data Sets

Three data sets representing distinct prediction tasks in drug discovery and development were used in this study: aqueous solubility (regression), mutagenicity (classification), and antiviral potency (regression). Data acquisition, filtering, and preprocessing procedures for each data set are described below. For the aqueous solubility and mutagenicity tasks, data were partitioned into disjoint training and test sets using a cross-validation protocol described below. For the potency task, the predefined data partitioning provided with the Antiviral Potency Challenge^47^ data set was used. Detailed data set statistics, including molecular property distributions, target value distributions, Bemis–Murcko scaffold statistics, and BRICS fragment statistics, are provided in the Supporting Information (Figures S1–S8 and Table S1).

#### 2.4.1 Aqueous Solubility

The aqueous solubility data set reported by Delaney^48^ was used. It comprises experimentally measured aqueous solubility values (in mol L^–1^) for 1144 compounds. The solubility values were transformed to logarithmic scale. The provided simplified molecular input line entry system (SMILES) strings were converted to nonisomeric canonical SMILES using RDKit.^44^ Duplicate entries were removed, resulting in a final data set of 1115 unique molecular structures with corresponding solubility values.

#### 2.4.2 Mutagenicity

For the mutagenicity classification task, a previously published data set^49^ containing 6512 compounds annotated with Ames test results was used. The Ames test is a standard experimental assay for assessing whether a chemical compound causes mutations.^50^ SMILES strings were converted to RDKit molecule objects and isomeric canonical SMILES representations and strings which could not be parsed by RDKit were discarded (6 invalid SMILES). Duplicate molecules based on the canonical isomeric SMILES were discarded (1 duplicate entry). Molecular fragments were generated using the BRICS algorithm.^43^ containing more than 16 fragments were excluded, resulting in the removal of 13 compounds from the data set. This threshold was chosen to ensure computational feasibility, as exact Shapley value computation scales factorially with the number of fragments. In addition, molecules with a very large number of fragments typically correspond to structurally complex, high-molecular-weight compounds that fall outside the domain of typical drug-like molecules considered in this study. The resulting data set comprised 6492 molecular structures.

#### 2.4.3 Antiviral Potency

For the potency regression task, we used the SARS-CoV-2 Mpro data set from the Antiviral Potency Challenge organized by ASAP (AI-driven Structure-enabled Antiviral Platform) Discovery x OpenADMET in 2025.^47^ The original data set consists of 1328 entries. Provided ChemAxon Extended SMILES (CXSMILES) were converted to canonical SMILES representations. As a consequence of this conversion, information regarding enantiomeric purity or racemic composition was lost. This limitation applies uniformly across all compounds. Compounds lacking experimentally determined pIC_50_ values were excluded. No additional compounds were removed during preprocessing in order to maintain comparability with the published challenge leaderboards. The final data set contained 1105 compounds of which 842 were used for training and 263 for testing.

### 2.5 Machine Learning Models and Hyperparameter Optimization

As representative models of classical and deep learning approaches in molecular machine learning, we consider a random forest (RF)^51^ and a graph convolutional network (GCN). This selection allows us to evaluate the proposed explanation framework across both tabular and graph-based molecular representations. The RF model was implemented using scikit-learn,^52^ while the GCN was implemented using PyTorch Geometric^53,54^ and PyTorch.^55^ For the RF model on the aqueous solubility and mutagenicity data sets, 5-fold nested cross-validation was employed, with hyperparameters optimized within each training fold using grid search combined with 5-fold internal validation. For the potency data set, the predefined train–test split was used, and hyperparameters were optimized on the training portion using grid search with 5-fold internal cross-validation. The corresponding search space is reported in Table 1. For the GCN model, the architecture consisted of multiple graph convolutional layers followed by a mean pooling layer. The pooled representation was subsequently passed to a fully connected network comprising multiple layers and a final output layer. Rectified linear unit (ReLU) activation functions and dropout were applied throughout the network. Model parameters were optimized using the Adam optimizer.^56^ For regression tasks, the mean squared error (MSE) loss function was used, whereas binary cross-entropy loss was employed for classification tasks. Hyperparameters of the GCN were optimized using random sampling from a predefined search space (Table 2). The best-performing hyperparameter configuration was then fixed and used consistently across all cross-validation folds. A total of 92, 88, and 100 configurations were evaluated for the solubility, mutagenicity, and potency data sets, respectively. The optimized GCN hyperparameters are summarized in Table 3. For both model classes, model selection was based on the root mean squared error for regression tasks and Matthews’ correlation coefficient^57^ for the classification task. The optimal hyperparameters were then used to retrain the model on the full training set of the respective fold. The resulting models were used for test set prediction and for computing fragment-level Shapley value attributions.

**Table 1:** Search Space for the Grid Search Employed for the RF Model for all Classification and Regression Tasks

parameter	search space
# estimators	25, 50, 100, 200, 400
minimum samples required at a leaf node	1, 2, 5, 10
minimum samples required to split	2, 3, 5, 10

**Table 2:** Search Space for the Random Search Employed for the GCN Model for all Classification and Regression Tasks

parameter	search space
# convolutional layers	2, 3, 4
# fully connected layers	2, 3, 4, 5
size fully connected layers	256, 512, 1024
hidden representation size (nodes)	64, 128, 256, 512
learning rate	1 × 10^–4^, 2 × 10^–4^, 1 × 10^–3^, 2 × 10^–3^
# maximum epochs	75, 100, 125, 150
batch size	4, 8, 16

**Table 3:** Hyperparameters of the GCN Model for all Employed Datasets

parameter	solubility	mutagenicity	SARS
# convolutional layers	3	3	3
# fully connected layers	3	2	3
size fully connected layers	1024	256	512
hidden representation size (nodes)	256	256	256
learning rate	0.0002	0.001	0.0002
# maximum epochs	125	125	150
batch size	8	8	8

### 2.6 Aggregation, Analysis, and Visualization of Attribution Scores

Fragment-level Shapley values were computed for all model predictions. For each data set, Shapley values were aggregated across identical fragment types by averaging the attribution values of all occurrences of a given fragment across molecules in which it appears. For visualization of individual predictions, fragment-level Shapley values were mapped onto the constituent atoms and bonds of each fragment. Contributions were visualized using diverging colormaps, where color intensity reflects the magnitude of the attribution and hue encodes its sign. For comparison with domain-agnostic explainability methods, additional attributions were generated using SHAP^18,19^ and GNNExplainer.^20^ For fingerprint-based models, SHAP values are defined at the level of individual bit positions. These were mapped to the central atoms of the corresponding atom environments and summed to obtain atom-level attributions prior to visualization. For graph-based models, GNNExplainer provides importance scores at the level of nodes. These scores are unsigned and not defined on an additive scale, and were therefore visualized using a sequential colormap. Due to these differences, GNNExplainer results are not directly comparable to fragment-level Shapley values in a quantitative sense. Molecular structures were visualized using RDKit.^44^ Additional figures were generated using matplotlib and seaborn. Data analysis and processing were performed using NumPy, SciPy, and pandas.

Code implementing the proposed fragment-level Shapley value framework, along with example scripts for applying the method to pretrained models, is made publicly available at https://github.com/jannik-roth/FragShapley (see Data Availability).

## 3 Results and Discussion

We first assess the predictive performance of the employed machine learning models, followed by an analysis of the interpretability of fragment-level Shapley values using the aqueous solubility and mutagenicity data sets. This is followed by a comparison with SHAP and GNNExplainer, two established explainability methods. Finally, we analyze the models’ learning characteristics and generalization capabilities on the potency prediction task.

### 3.1 Model Performance

First, we assess predictive performance to ensure that the models capture relevant structure–property relationships. For the regression data sets (aqueous solubility and SARS potency), the root mean squared error (RMSE), mean absolute error (MAE), and coefficient of determination (*R* ^2^) were evaluated. For the aqueous solubility data set, the GCN model achieves slightly higher predictive performance than the RF model, with a mean *R* ^2^ value of 0.73 compared to 0.68 for the RF model (Table 4, Figure S9). In the original publication of the data set,^48^ a multiple linear regression model achieved an *R* ^2^ of 0.72, which is comparable to the performance observed here. For the SARS potency data set, *R* ^2^ values of 0.61 (RF) and 0.42 (GCN) were obtained (Table 4, Figure S10). The corresponding MAE values of 0.63 (RF) and 0.79 (GCN) are lower than those of the baseline linear model reported for this data set (MAE = 0.98).^58^

**Table 4:** Performance Metrics for the RF and GCN Models on the Regression Datasets (Solubility and SARS)^a^

data set	model	RMSE	MAE	*R* ^2^
solubility	RF	1.162 ± 0.076	0.867 ± 0.043	0.684 ± 0.055
	GCN	1.071 ± 0.105	0.817 ± 0.090	0.726 ± 0.079
SARS	RF	0.828	0.632	0.614
	GCN	1.012	0.792	0.423

a For the solubility dataset, five-fold cross-validation was performed and the reported values are the mean and one standard deviation. For the SARS dataset, the provided train and test partitioning was employed.

For the mutagenicity classification task, both models achieve strong predictive performance, with ROC-AUC values of 0.88 (RF) and 0.85 (GCN), which fall within or slightly above the range reported in the original publication^49^ of 0.79 to 0.86 (Table 5).

**Table 5:** Performance Metrics for the RF and GCN Models on the Mutagenicity Dataset^a^

model	ROC-AUC	precision	recall
RF	0.880 ± 0.011	0.821 ± 0.013	0.821 ± 0.027
GCN	0.846 ± 0.013	0.773 ± 0.009	0.795 ± 0.028

a Reported are the mean and one standard deviation across five-fold cross-validation.

Overall, the employed models achieve comparable predictive performance on the solubility and mutagenicity tasks, while performance on the SARS potency data set is lower and reflects the increased difficulty of this prediction task. Nevertheless, the models capture sufficient structure–property signal across all data sets to support the subsequent analysis of fragment-level explanations. Nevertheless, the models achieve predictive performance above simple baseline models or within previously reported ranges for the corresponding data sets, indicating that they capture nonrandom structure–property relationships. The subsequent attribution analyses should therefore be interpreted as explanations of the learned model behavior rather than as direct evidence of causal structure–property relationships.

### 3.2 Fragment-Level Shapley Values Reproduce Known Solubility Trends

Aqueous solubility provides a well-characterized structure–property relationship and therefore serves as a suitable test case for evaluating whether fragment-level Shapley values recover established chemical trends.^48,59^ Interpretable explanations are particularly valuable in this context for guiding compound optimization.^23^ For organic compounds, the empirical principle of “like dissolves like” is commonly applied to rationalize solubility behavior: polar moieties tend to increase solubility in polar solvents, whereas nonpolar moieties favor solubility in nonpolar solvents.^60^

To empirically assess whether the employed models capture this principle, we analyzed the fragment-level explanations for the aqueous solubility prediction task. In the representative examples shown in Figure 1A, aromatic and aliphatic fragments exhibit negative Shapley contributions, whereas polar functional groups, such as carbamides, hydroxyl groups, and amides, exhibit positive contributions to aqueous solubility. The data set-wide aggregation of fragment-level Shapley values (Figure 1B) further confirms these trends: aromatic and aliphatic fragments show the strongest negative contributions to solubility, heteroatom-containing fragments such as amines and ethers exhibit comparatively small effects, and carbonyl-containing fragments display the largest positive contributions to aqueous solubility. Subtle differences in alkyl chain length are also resolved. For example, the mean contribution of the *n*-butyl fragment is more negative than that of the *n*-propyl fragment.

**Figure 1:**
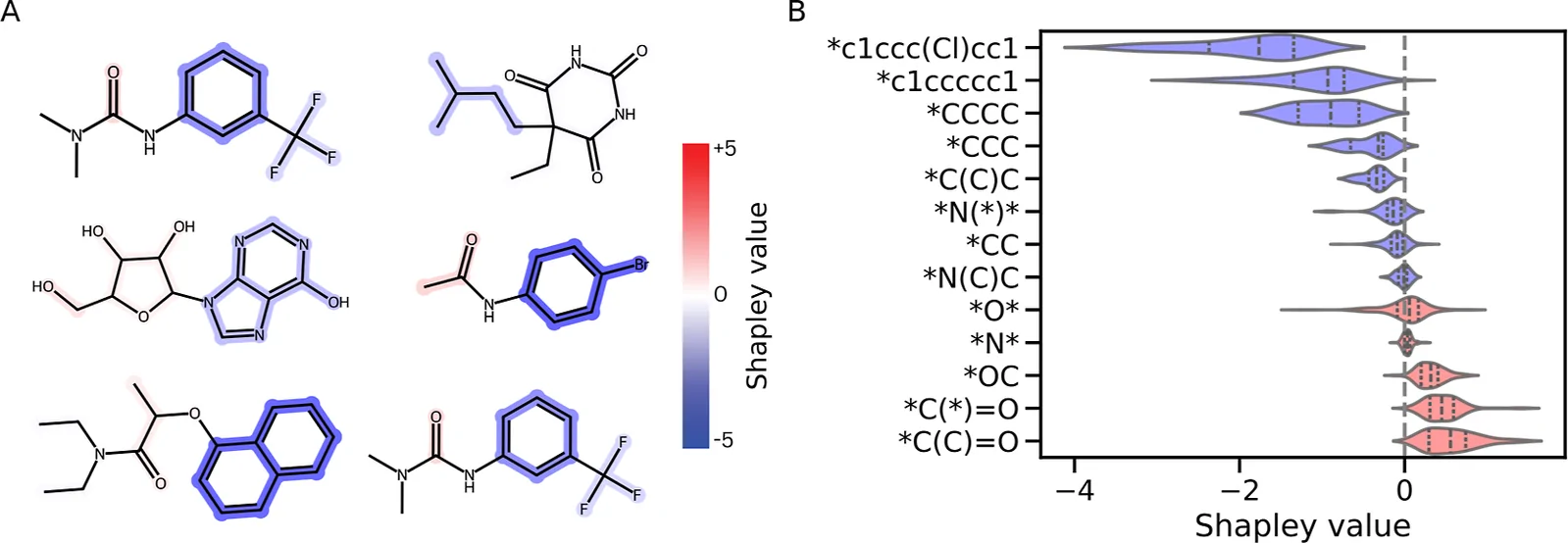
A) Fragment-level Shapley value visualization for representative RF predictions on the aqueous solubility data set. (B) Distribution of Shapley values for fragments occurring at least 20 times. Violin plots show median and interquartile range. The vertical dashed line indicates zero contribution.

Beyond qualitative consistency, the additive property of Shapley values enables a quantitative assessment of fragment contributions. The sum of all Shapley values equals the model prediction up to a constant baseline, such that contributions are expressed on the same scale as the predicted target property. To illustrate this behavior, a 1,3,5-trisubstituted cyclohexane scaffold was systematically decorated with fragments derived from compounds in the data set, and the aqueous solubility of the resulting molecules was predicted. Comparing the predicted solubility values to the sum of the mean fragment-level Shapley values associated with the substituents (Figure 2) reveals a positive correlation (Pearson *r* = 0.80). This example serves as an illustrative case demonstrating how fragment contributions combine to approximate the model output.

**Figure 2:**
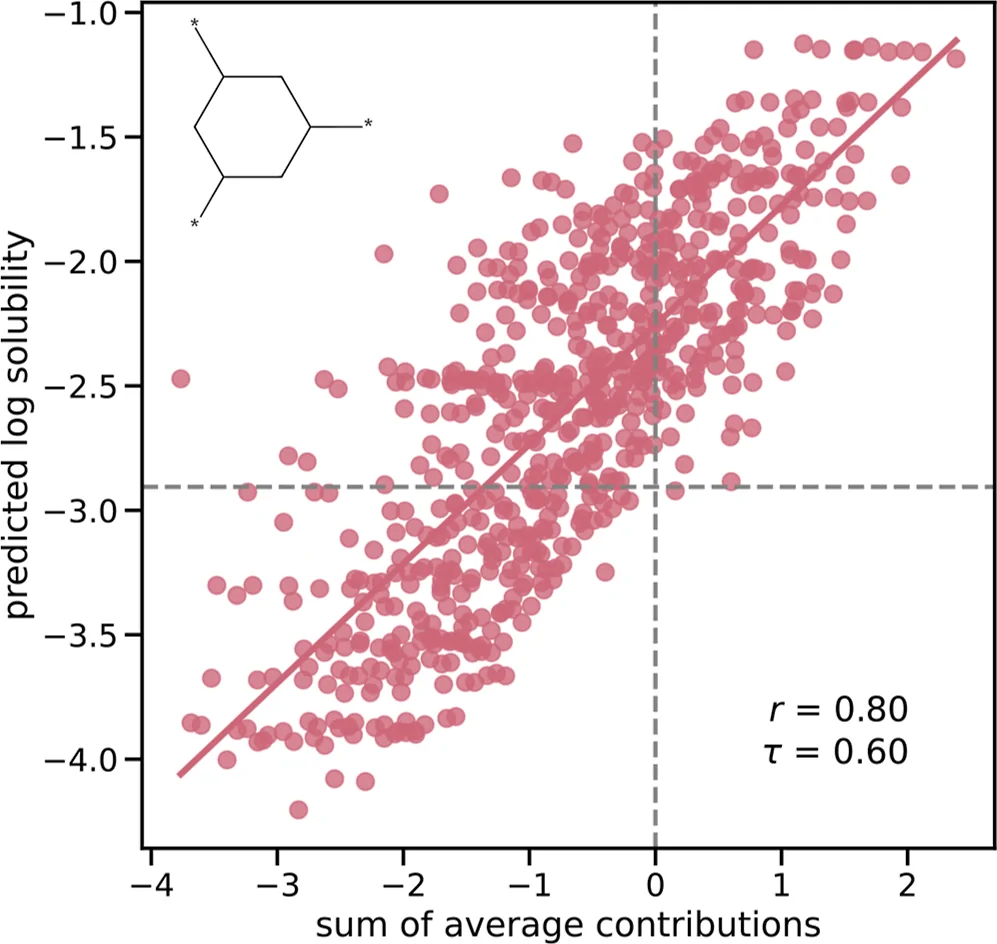
Scatter plot of predicted aqueous solubility (RF model) versus the sum of mean fragment-level Shapley values for R-groups on a 1,3,5-trisubstituted cyclohexane scaffold. R-groups occur at least three times in the data set. The regression line indicates the central trend. Pearson (*r*) and Kendall (τ) correlation coefficients are reported and are statistically significant (*p* < 1 × 10^–10^).

To further assess whether fragment-level Shapley values capture meaningful structure–property relationships beyond qualitative trends, we analyzed analogue series defined by Bemis—Murcko scaffolds.^61^ For each series, the predicted solubility values were compared to the sum of the average Shapley contributions of the corresponding nonscaffold fragments. The largest analogue series, comprising compounds containing a phenyl scaffold, showed strong correlations between predicted solubility and the summed fragment contributions for both the RF (Pearson *r* = 0.79) and GCN (Pearson *r* = 0.87) models (Figure S11 and Table S2). Similar correlations were observed for additional analogue series (Table S2). These results indicate that fragment-level Shapley values capture substituent effects that are consistent across molecules sharing a common core structure and therefore recover meaningful trends within analogue series.

To assess the sensitivity of the method to the baseline choice, we performed an additional analysis on the RF model for the aqueous solubility data set (see Supporting Information, Sensitivity Analysis on the Empty Coalition Value). The original empty-coalition prediction (ν­(Ø) = −1.06) was replaced by an alternative baseline value of 0, and fragment-level Shapley values were recomputed. Individual fragment attributions remained highly consistent between the two settings (Pearson *r* = 0.98, Kendall τ = 0.85, Figure S12). We further compared data set-level average fragment contributions and observed similarly strong agreement (Pearson *r* = 0.99, Kendall τ = 0.85, Figure S13). These results indicate that the principal chemical trends and relative fragment rankings are largely invariant to reasonable changes in the baseline definition, despite the expected shift in absolute attribution values.

Although aqueous solubility itself arises from complex, nonadditive physicochemical interactions, the model learns an approximately additive representation of fragment contributions. The observed correspondence therefore indicates that fragment-level Shapley values provide a meaningful approximation of the model output, enabling direct comparison of fragment effects across molecules. This facilitates the identification of favorable and unfavorable substituents and supports rational solubility optimization. Taken together, these observations indicate that the learned structure–property relationships are consistent with established chemical intuition regarding aqueous solubility.

### 3.3 Identification of Toxicophores and Shapley Value-Guided Molecular Optimization

Mutagenicity prediction is commonly rationalized using the concept of toxicophores, i.e., substructures associated with increased mutagenic risk. This makes mutagenicity a suitable test case for evaluating whether fragment-level Shapley values recover chemically meaningful structure–activity relationships. To this end, we generated predictions and corresponding explanations for the entire mutagenicity data set.

In representative correctly classified mutagenic compounds (Figure 3A), fragment-level Shapley values highlight specific substructures or even individual fragments that strongly contribute to the prediction. This behavior is consistent with the toxicophore concept, where the presence of a single substructure can dominate mutagenicity risk. Data set-wide aggregation of fragment-level Shapley values (Figure 3B) reveals consistent trends across both RF and GCN models. In both cases, fragments with the highest average positive Shapley values include aromatic nitro groups, epoxides, and aromatic amines, although their exact order differs slightly. In addition, the RF model assigns high contributions to α, β-unsaturated aldehydes and acid chlorides, whereas the GCN model highlights aliphatic halogens. These fragments correspond to those with the highest average positive Shapley values among frequently occurring fragments and match substructures previously reported as toxicophores.^62^ Taken together, these results indicate that the models predominantly rely on a limited set of chemically well-defined substructures to drive mutagenicity predictions, consistent with established toxicophore concepts.

**Figure 3:**
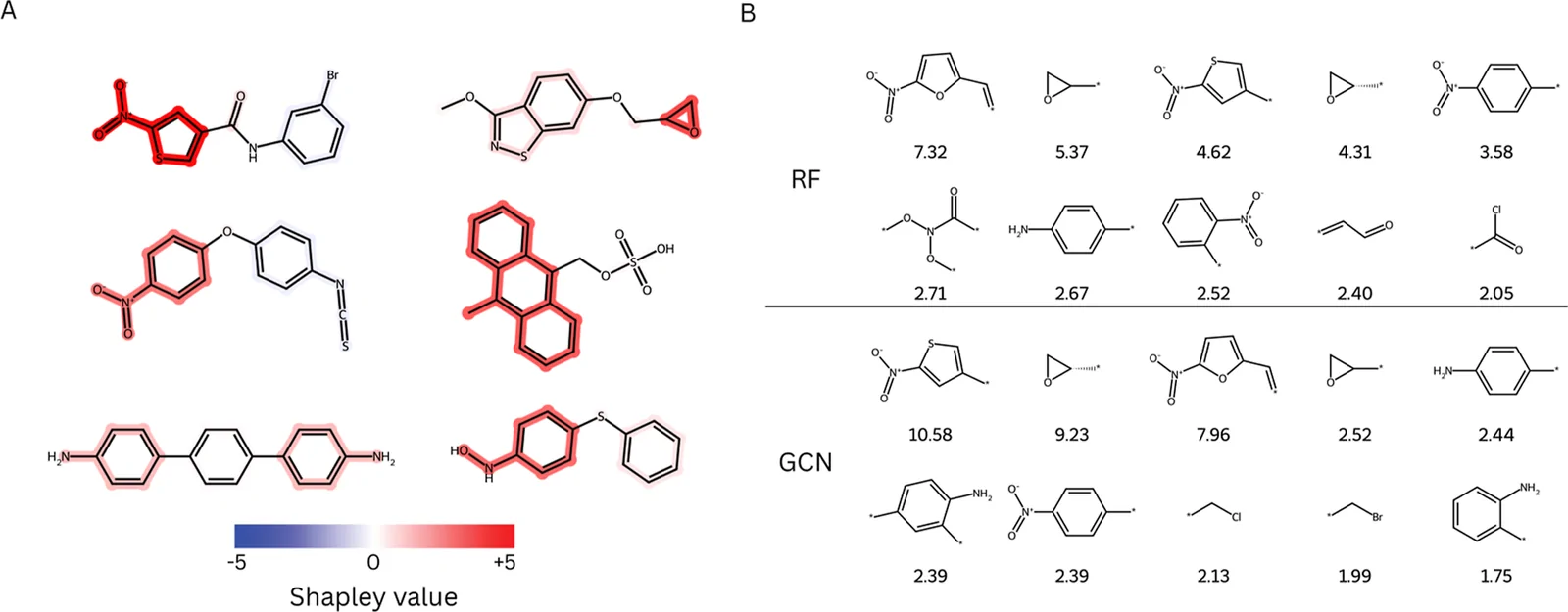
(A) Fragment-level Shapley value visualization for representative correctly predicted mutagenic compounds (GCN model). (B) Fragments with the highest positive mean Shapley values across the mutagenicity data set (occurrence ≥20), shown for RF (top) and GCN (bottom).

To further assess the extent to which the models rely on known toxicophores, we analyzed the distribution of established toxicophore motifs reported by Kazius et al.^62^ across the data set and evaluated model performance separately for compounds containing and lacking such motifs. While compounds containing at least one known toxicophore were strongly enriched in mutagenic compounds (77% mutagenic), compounds without known toxicophores were predominantly nonmutagenic (76% nonmutagenic) (Table S4). However, both RF and GCN models achieved comparable ROC-AUC values on the toxicophore-containing and nontoxicophore subsets (Table S5). These findings indicate that known toxicophores contribute substantially to model performance, but suggest that the models are not solely performing toxicophore matching and instead capture additional structural information associated with mutagenicity beyond the presence of established toxicophore motifs.

Beyond identifying relevant substructures, fragment-level Shapley values enable a quantitative assessment of how individual fragments influence model predictions. This provides a direct link between model interpretation and molecular optimization, where fragments with large positive contributions can be targeted for modification. The fragment-level explanations therefore enable systematic modification of fragments associated with high predicted mutagenicity (Figure 4A). After replacement of the identified fragment with a group exhibiting a negative mean Shapley value, all modified compounds in these examples are predicted as nonmutagenic by the GCN model. In each case, replacement of the fragment with the highest positive Shapley value results in a reversal of the model’s classification.

**Figure 4:**
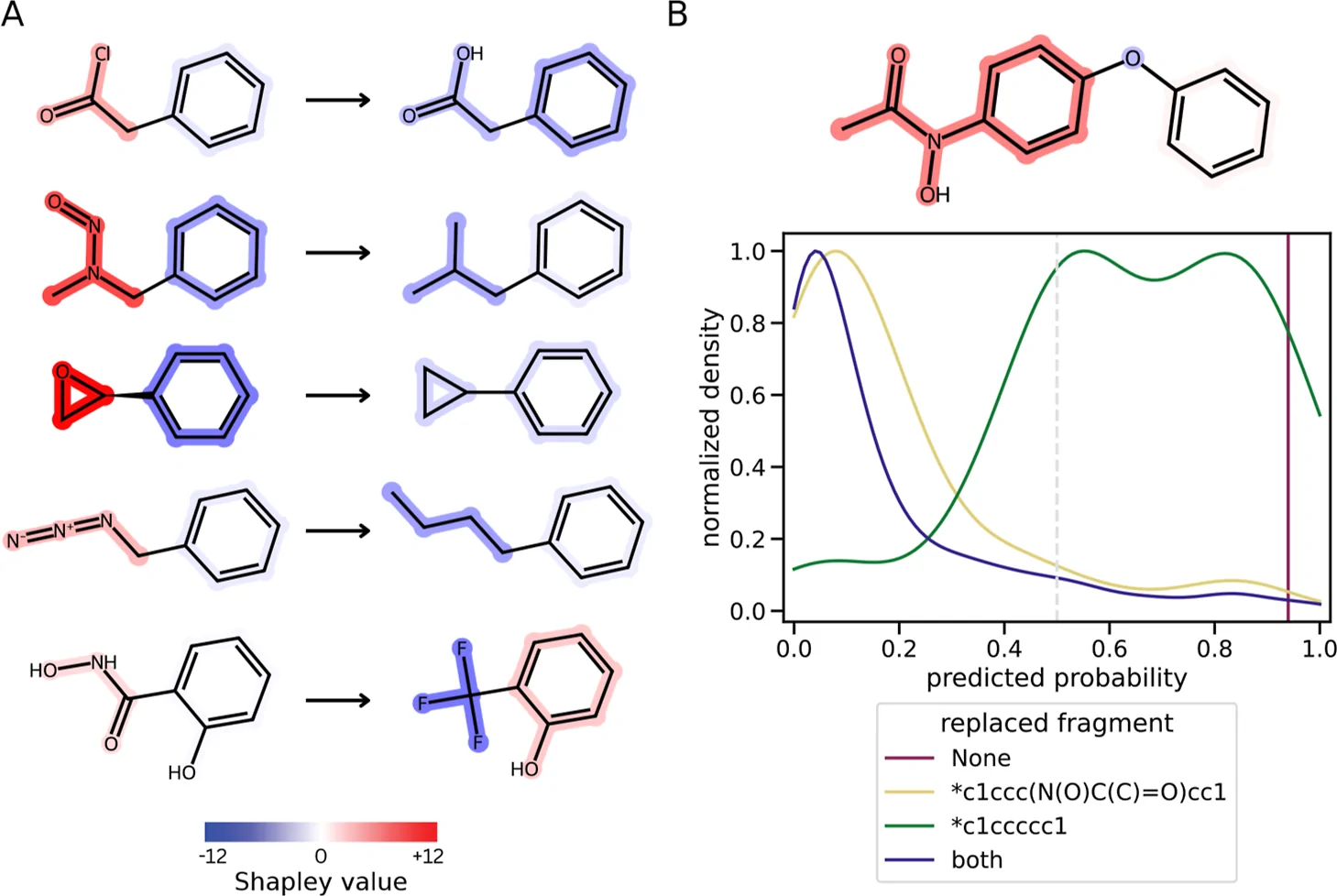
(A) Shapley value-guided fragment replacement for compounds predicted as mutagenic (GCN model). Left: original molecules; right: modified molecules after replacing fragments with high positive Shapley values by fragments with negative mean Shapley values (occurrence ≥3), resulting in nonmutagenic predictions. (B) Fragment replacement for a compound with multiple mutagenicity-contributing fragments. Top: original molecule. Bottom: kernel density estimates of predicted mutagenicity probabilities after replacing individual fragments or both simultaneously. The vertical solid line indicates the original prediction. The dashed line marks the decision threshold.

To further investigate this behavior, we extended the analysis to a more complex structure containing two contributing fragments (Figure 4B). In the original molecule (Figure 4B, top), two fragments are identified as contributors to a mutagenic prediction: an *N*-Hydroxy-*N*-phenylacetamide moiety (SMILES: *c1ccc­(N­(O)­C­(C)=O)­cc1) with a Shapley value of 5.8 and a phenyl group (SMILES: *c1ccccc1) with a Shapley value of 0.6. While phenyl groups are not generally considered toxicophores, the model assigns a small positive contribution, suggesting that fragment contributions depend on their structural context rather than solely on their identity. Upon replacement of fragments contributing toward a mutagenic prediction with fragments exhibiting negative mean Shapley values, the resulting compounds were evaluated using the GCN model. The original molecule is predicted as mutagenic with a probability of 0.94. Replacing the phenyl group shifts predicted probabilities to lower values; however, 81% of the resulting compounds remain classified as mutagenic, indicating that this modification alone is insufficient. In contrast, replacement of the *N*-Hydroxy-*N*-phenylacetamide fragment leads to 92% of generated compounds being classified as nonmutagenic. Replacing both fragments yields a similarly high fraction of nonmutagenic predictions (91%). These results demonstrate that fragment-level Shapley values not only identify relevant substructures but also rank their relative importance, enabling prioritization of modifications in molecules containing multiple contributing fragments.

Overall, fragment-level Shapley values capture chemically meaningful structure–mutagenicity relationships and enable systematic identification of substructures associated with increased predicted mutagenicity. In addition, the ranking of fragment contributions provides actionable guidance for targeted molecular modification. However, these assessments reflect model predictions and should not be interpreted as direct measures of experimental toxicity. Furthermore, the analysis is based on Ames test data and does not capture broader aspects of human toxicity.

### 3.4 Comparison to Existing Explainability Methods

To assess the effect of computing Shapley attributions on chemically meaningful fragments, we compare our approach to two widely used posthoc explainability approaches on the aqueous solubility task. For the RF model, we generated atom-level attributions using SHapley Additive exPlanations (SHAP),^18,19^ which assigns importance values to fingerprint features corresponding to atom environments. For the GCN model, we computed explanations using GNNExplainer,^20^ which identifies important subgraphs via node and edge importance masks.

Figure 5A compares fragment-level Shapley attributions with atom-level SHAP explanations for the RF model. While the higher resolution of the SHAP approach can be useful for identifying localized effects, it often leads to fragmented attribution patterns that are more difficult to interpret chemically. In particular, SHAP values can vary substantially between atoms belonging to the same functional group or fragment, or concentrate on single atoms (e.g., the aromatic chlorine atom in the second row), making it less straightforward to relate these attributions to chemically meaningful substructures or to translate them into modification strategies. At the data set level, SHAP-based aggregation (Figure 5B) yields trends broadly consistent with the fragment-based analysis (Figure 1B): carbonyl- and heteroatom-containing environments tend to contribute positively to aqueous solubility, whereas aromatic and aliphatic environments contribute negatively. Here, atom environments refer to the local neighborhoods encoded by the fingerprint representation. However, some environments exhibit counterintuitive average effects (e.g., aromatic environments with positive mean SHAP values). Because multiple, overlapping atom environments contribute to each molecule, aggregation across the data set is less direct than for nonoverlapping fragment-based attributions, complicating interpretation and comparison.

**Figure 5:**
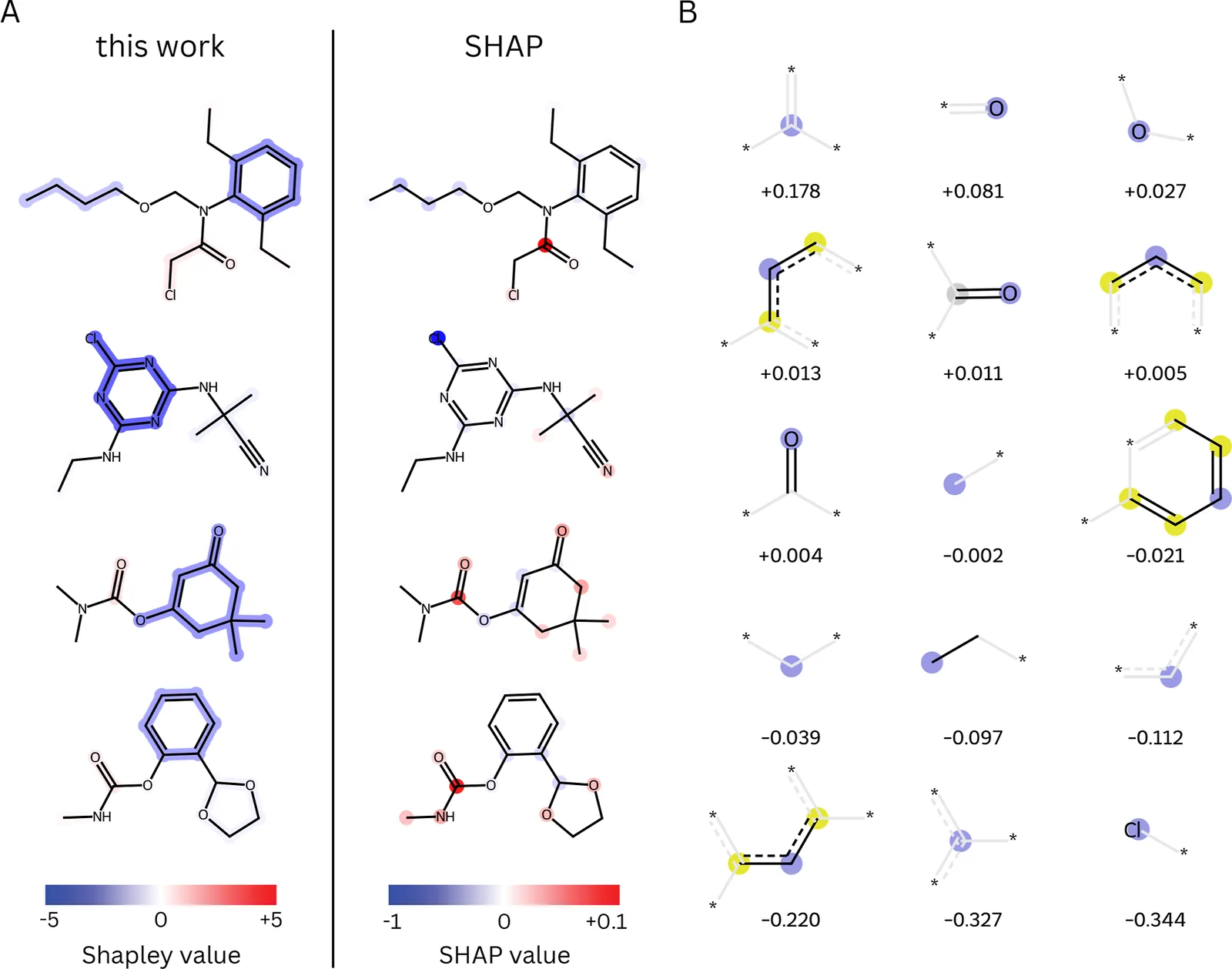
(A) Comparison of fragment-level Shapley attributions (left) and atom-level SHAP explanations (right) for the RF model on the solubility data set. Note that color scales differ between methods. (B) Most frequent atom environments with their mean SHAP values (computed over molecules in which the environment is present).

For graph-based models, we compare the proposed approach to GNNExplainer, which learns sparse node and edge masks to identify substructures that are most relevant for a model’s prediction. Representative examples (Figure 6) highlight key differences between the two approaches. Fragment-level Shapley values provide signed attribution values, enabling direct differentiation between contributions that increase or decrease the predicted property. In contrast, GNNExplainer yields unsigned importance scores that indicate relative relevance but do not encode the direction of the effect. Furthermore, these importance scores are not defined on an additive scale, which limits quantitative interpretation both within and across molecules. In our examples, GNNExplainer importance scores also fluctuate strongly across atoms within the same functional group, limiting interpretability for downstream design. In contrast, fragment-level Shapley values yield structurally coherent, fragment-consistent attributions that are easier to interpret chemically and to act upon in molecular optimization. Unlike SHAP, GNNExplainer does not naturally yield data set-level importance statistics, limiting direct comparison of model-wide trends.

**Figure 6:**
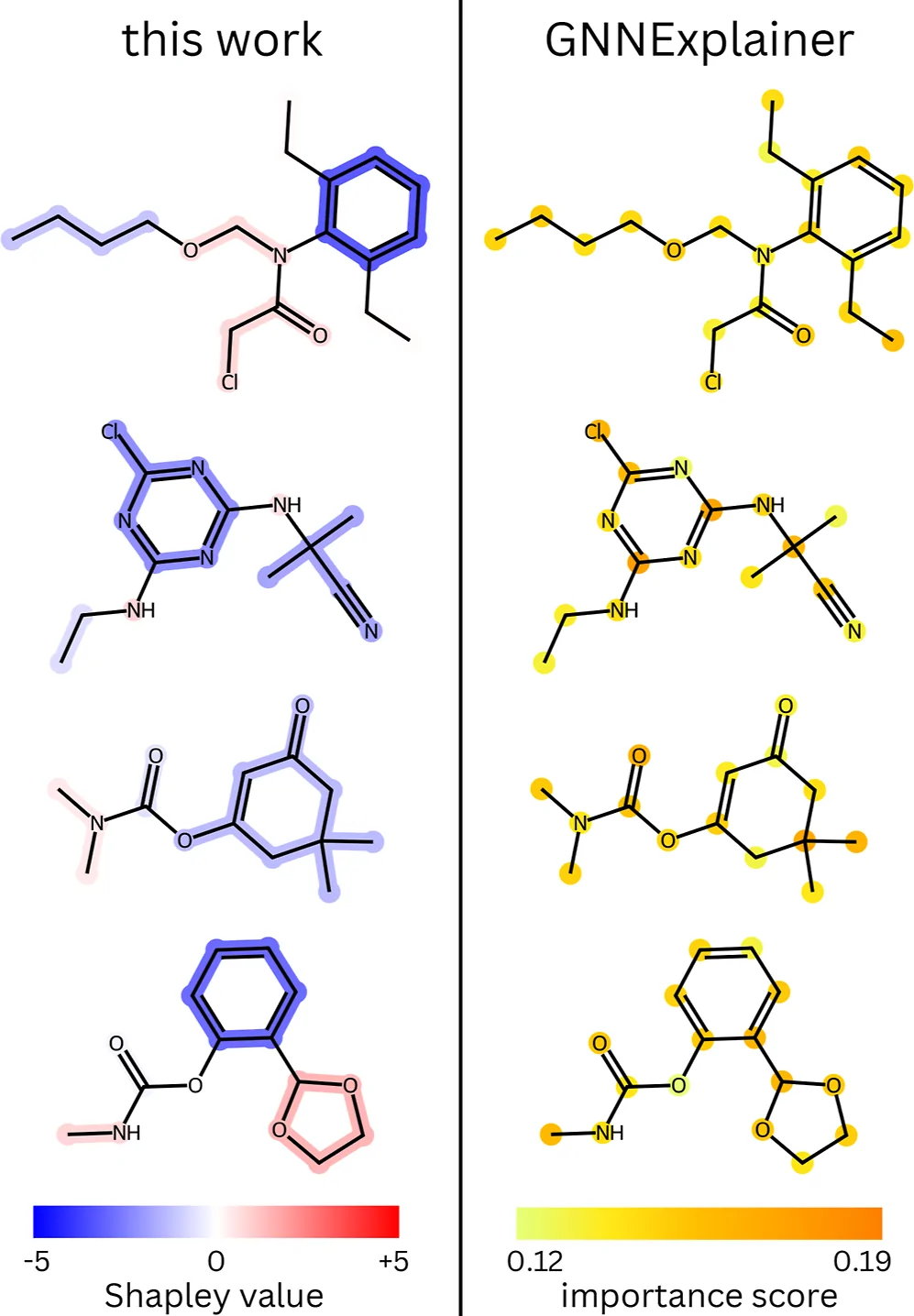
Visual comparison of fragment-level Shapley attributions (left) and GNNExplainer importance scores (right) for the GCN model on the solubility data set. Shapley values are signed, whereas GNNExplainer scores are unsigned and shown using a sequential colormap.

Overall, the comparison suggests that fragment-level Shapley values provide more coherent and chemically interpretable attributions in this setting. In addition, the additive nature of the Shapley framework enables quantitative analyses of fragment contributions that are difficult to perform using the compared explanation methods.

While atom-level and subgraph-based methods can offer higher-resolution insights or highlight relevant regions of a molecule, fragment-level attributions facilitate chemical reasoning and support downstream tasks such as molecular optimization.

### 3.5 Interpreting Learning Trends and Generalization in Potency Prediction

Among the three data sets considered in this study, the SARS potency prediction task proved to be the most challenging for both the RF and GCN models in terms of predictive performance. At the same time, the models retain sufficient predictive signal (see Model Performance) to make this data set informative for analyzing how predictions are formed and where limitations arise. To evaluate the learned prediction patterns and identify model-wide trends, fragment-level Shapley values were generated for all compounds in the test set. Representative predictions of the GCN model together with their Shapley value mappings (Figure 7A) reveal that predictions are primarily driven by a central core structure, while substituents decorating this core have comparatively limited influence on the predicted potency. This behavior contrasts with the solubility and mutagenicity data sets, where smaller fragments, including simple substituents, often contributed substantially to the final prediction. This observation is further supported by aggregation of fragment-level Shapley values across the data set (Figure 7B). For both models, the fragments with the highest average Shapley values correspond to closely related core structures that differ only by minor substitutions (e.g., chlorine or fluorine atoms). Mean Shapley values of up to 2 are observed for these cores, corresponding to an approximately 100-fold increase in predicted potency. Fragments ranked below these core structures, such as ethylene, isopropyl, ether, or (secondary) amine groups, are more generic and occur frequently in drug-like molecules. While some of these fragments exhibit relatively high average Shapley values (e.g., trifluoromethyl with 1.58 for the GCN model), their widespread occurrence limits their discriminatory power. Overall, the prediction of potent compounds appears to be dominated by the presence of specific core structures, with substituent effects playing a secondary role.

**Figure 7:**
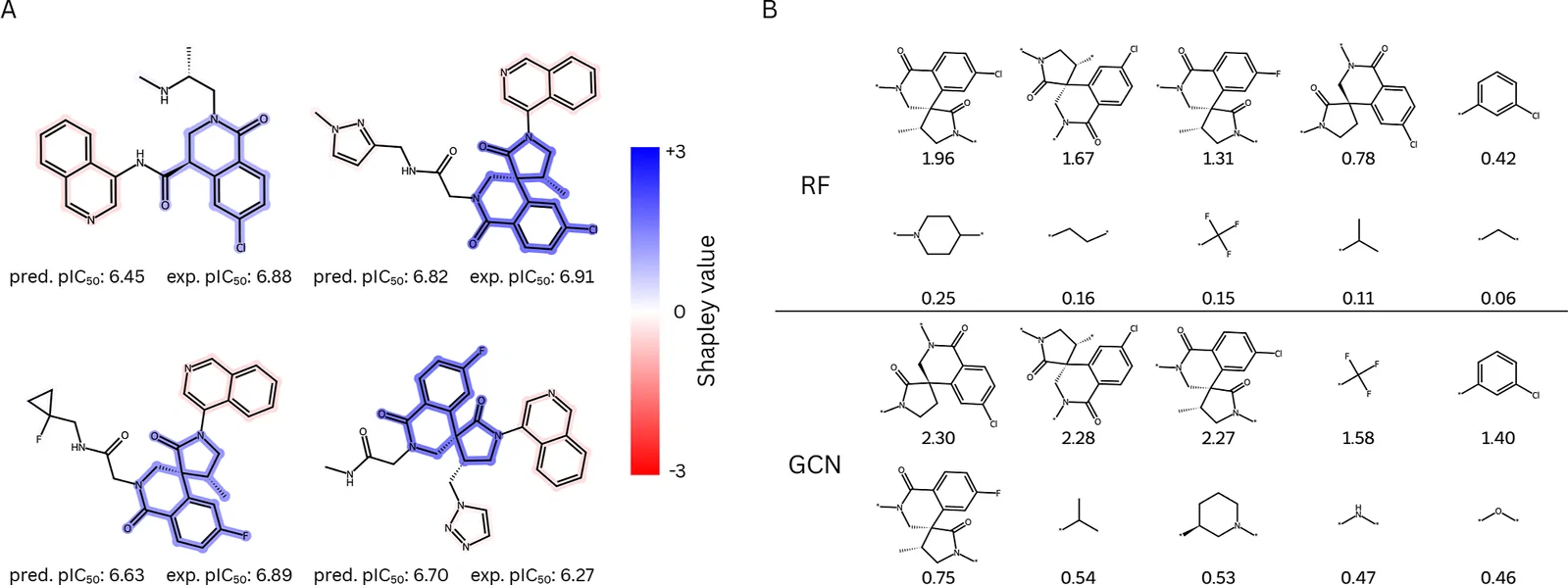
(A) Fragment-level Shapley attributions for representative predictions of the GCN model on the SARS data set. Blue indicates contributions toward higher predicted pIC_50_ values, red toward lower values; predicted and experimental pIC_50_ values are shown below each molecule. (B) Fragments with the highest positive average Shapley values across the SARS data set for RF (top) and GCN (bottom). Only fragments occurring at least five times are included. The average Shapley values are reported below each structure.

While this behavior is consistent with the general principle that structurally similar compounds tend to exhibit similar biological activity, it also highlights a potential limitation of the learned models. The dominance of core structures in the fragment-level Shapley value analysis suggests that predictions are largely driven by scaffold similarity rather than by more fine-grained structure–activity relationships governing binding strength. In other words, the models appear to rely heavily on recognizing chemotypes represented in the training data. In this study, the predefined train–test split was retained to ensure comparability with previously reported models. However, alternative partitioning strategies, such as temporal splits,^63^ scaffold-based splits,^64^ or clustering-based splits,^65^ may provide a more stringent assessment of prospective performance and scaffold-level generalization. The fragment-level Shapley analysis thus serves as a diagnostic tool: it reveals that more accurate predictions are predominantly associated with known core structures and highlights a limited applicability domain for compounds containing novel scaffolds. In this context, Shapley values provide insight not only into individual predictions but also into model learning behavior and generalization characteristics.

Taken together, these results demonstrate that fragment-level Shapley values not only provide chemically meaningful explanations but also enable a practical assessment of model behavior, including limitations in generalization and applicability across chemical space.

### 3.6 Methodological Considerations and Limitations

While fragment-level Shapley values provide interpretable and chemically meaningful explanations, several methodological aspects merit further discussion. First, the approach relies on a predefined molecular fragmentation scheme. In this work, BRICS fragmentation was employed due to its retrosynthetic interpretability and its generation of nonoverlapping fragments, which enables unambiguous attribution of contributions to distinct substructures. In contrast, feature-based representations such as fingerprint environments may overlap substantially, complicating interpretation. Nevertheless, alternative fragmentation strategies may lead to different attribution patterns, and the choice of fragmentation therefore introduces an additional design parameter that can influence the granularity and chemical relevance of the resulting explanations, which represents a trade-off between interpretability and structural resolution. Second, the proposed framework assumes that molecules can be decomposed into a small number of fragments, which is typically valid for drug-like compounds but may become limiting for larger or highly complex structures. In such cases, the number of fragment combinations increases rapidly, restricting the feasibility of exact Shapley value computation. For such systems, sampling-based approximation strategies may provide a practical alternative while retaining the fragment-level interpretation, albeit at the cost of exactness.

Across the three prediction tasks, both RF and GCN models recovered the same principal qualitative trends, including known solubility relationships, established mutagenicity toxicophores, and scaffold-dominated behavior for the SARS potency data set, although attribution magnitudes and fragment rankings differed between model classes. This suggests that the dominant chemical trends are not specific to a single model architecture, although the resulting fragment attributions remain specific to the behavior learned by each individual model.

More generally, the proposed framework requires defining model inputs corresponding to arbitrary subsets of molecular fragments. Such fragment coalitions do not necessarily correspond to chemically valid molecules. For ECFP-based models, fragment coalitions are represented as subsets of atom-environment features derived from the original molecule. For graph-based models, fragment coalitions may yield disconnected graphs or boundary atoms whose feature vectors reflect their original molecular context. Consequently, fragment coalitions should be interpreted as representation-level perturbations of the original molecular representation rather than physically realizable molecular structures. This limitation is not unique to the proposed method but reflects a general challenge when applying perturbation-based attribution methods to molecular machine learning models.

Beyond computational considerations, fragment-level attributions, while more interpretable than feature-level explanations, remain model-dependent and reflect learned correlations rather than causal structure–property relationships governing molecular behavior. This becomes particularly relevant in settings where model performance is limited, as observed for the SARS potency task. In such cases, Shapley values remain informative as a diagnostic tool for identifying learned patterns and potential biases, but the resulting explanations should be interpreted with appropriate caution. Finally, fragment-level Shapley values complement rather than replace existing explainability methods. While they provide signed, additive, and chemically interpretable contributions, atom-level approaches such as SHAP or subgraph-based methods such as GNNExplainer can offer higher-resolution (atom-level) or subgraph-focused perspectives on model behavior. Combining multiple explanation methods may therefore provide a more comprehensive understanding of model predictions.

## 4 Conclusions

In this study, we introduced a framework for computing exact, fragment-level Shapley values for molecular machine learning. By decomposing molecules into a small number of chemically meaningful fragments, the Shapley value formalism can be applied without sampling-based approximations or feature imputation, yielding additive attribution values on the same scale as the model output. This task-specific adaptation offers practical advantages compared to general-purpose XAI methods: explanations are chemically intuitive and directly actionable at the fragment level. Additionally, the method operates post hoc on trained models, does not require specialized architectures, and is demonstrated for two widely used molecular representations, namely extended-connectivity fingerprints and molecular graphs, while being in principle applicable to other representations that allow construction of inputs for fragment subsets. Across three representative prediction tasks, fragment-level Shapley values provided chemically coherent and practically useful explanations. For aqueous solubility, the method reproduced well-established solubility trends and enabled model-wide analyses supporting rational solubility tuning through fragment substitution. For mutagenicity, fragment-level attributions identified known toxicophores and guided targeted fragment replacement. In the SARS potency task, despite lower predictive performance compared to simpler baseline models, the analysis revealed scaffold-dominated prediction behavior, providing insight into model learning characteristics and indicating limited generalization to structurally novel compounds. Comparisons with SHAP and GNNExplainer demonstrated that fragment-level attributions improve interpretability and actionability, particularly for downstream molecular design tasks. Overall, these results demonstrate that adapting a principled explainability framework to domain-specific molecular representations can enhance interpretability and diagnostic power. Although this specialization reduces some generality relative to fully domain-agnostic XAI methods, it enables explanations that align more closely with chemical reasoning and support informed decision-making in molecular design. More broadly, the approach underscores the value of integrating domain knowledge into explainability methods to bridge the gap between predictive performance and practical utility in drug discovery.

## Supplementary Material



## Notes

Molecular property distributions, target value distributions, Bemis–Murcko scaffold and BRICS fragment statistics for all data sets; additional regression performance results; analogue-series analysis of fragment-level Shapley values for aqueous solubility; sensitivity analysis of the empty-coalition baseline; and analysis of toxicophore occurrence and model performance for mutagenicity prediction (PDF)

## Notes

**Data and Software Availability** All data sets used in this study are publicly available. Data processing, model implementation, model checkpoints and data analysis scripts are available at https://github.com/jannik-roth/FragShapley and at 10.5281/zenodo.21001027.
