# Supplementary material for: Chemically Interpretable Explanations for Molecular Property Prediction via Fragment-Level Shapley Values
Source: J Chem Inf Model. 2026 Aug 14;66(17):10661–73. doi: 10.1021/acs.jcim.6c01425 (PMC13580134; doi:10.1021/acs.jcim.6c01425)
Supplement: Supplementary file 1 [file ci6c01425_si_001.pdf]

Supporting Information for:

Chemically Interpretable Explanations for Molecular Property Prediction via Fragment-Level Shapley Values

Jannik P. Roth*[1,2,3]

[1]Mulliken Center for Theoretical Chemistry, University of Bonn, 53115 Bonn, Germany
[2]Life and Medical Sciences (LIMES) Institute, University of Bonn, 53115 Bonn, Germany
[3]Bonn Center for Mathematical Life Sciences, University of Bonn, 53115 Bonn, Germany

*Email: jproth@uni-bonn.de

Contents

Characterization of Datasets **S1**
Aqueous Solubility S2
Mutagenicity S4
Antiviral Potency S5

Predictive Performance on Regression Tasks **S6**

Analysis of Solubility Trends across Analogue Series **S7**

Sensitivity Analysis on the Empty Coalition Value **S8**

Analysis of Reliance on Toxicophores for Mutagenicity Prediction **S10**

Characterization of Datasets

To further characterize the datasets used in this study, we analyzed the distributions of BRICS fragments[1], Bemis—Murcko scaffolds[2], target values, and selected physico-chemical properties. Analogue-series statistics were computed only for molecules yielding a Bemis—Murcko scaffold.

Table S1: Dataset statistics including the number of Bemis–Murcko scaffolds (BM), average analogue-series size (AS), total and unique BRICS fragments, average number of BRICS fragments per molecule, and the number of compounds yielding a single BRICS fragment.

dataset	n	BM	avg. AS	BRICS	unique	avg./mol	single
aqueous solubility	1115	268	2.99	2510	999	2.25	568
SARS	1105	481	2.30	5250	704	4.75	0
mutagenicity	6492	1522	3.53	16278	5182	2.51	2792

The proportion of molecules yielding a single BRICS fragment varies substantially across datasets. While all compounds in the antiviral potency dataset decompose into multiple fragments, approximately half of the

molecules in the aqueous solubility and mutagenicity datasets yield a single fragment and therefore result in a trivial Shapley decomposition.

Aqueous Solubility

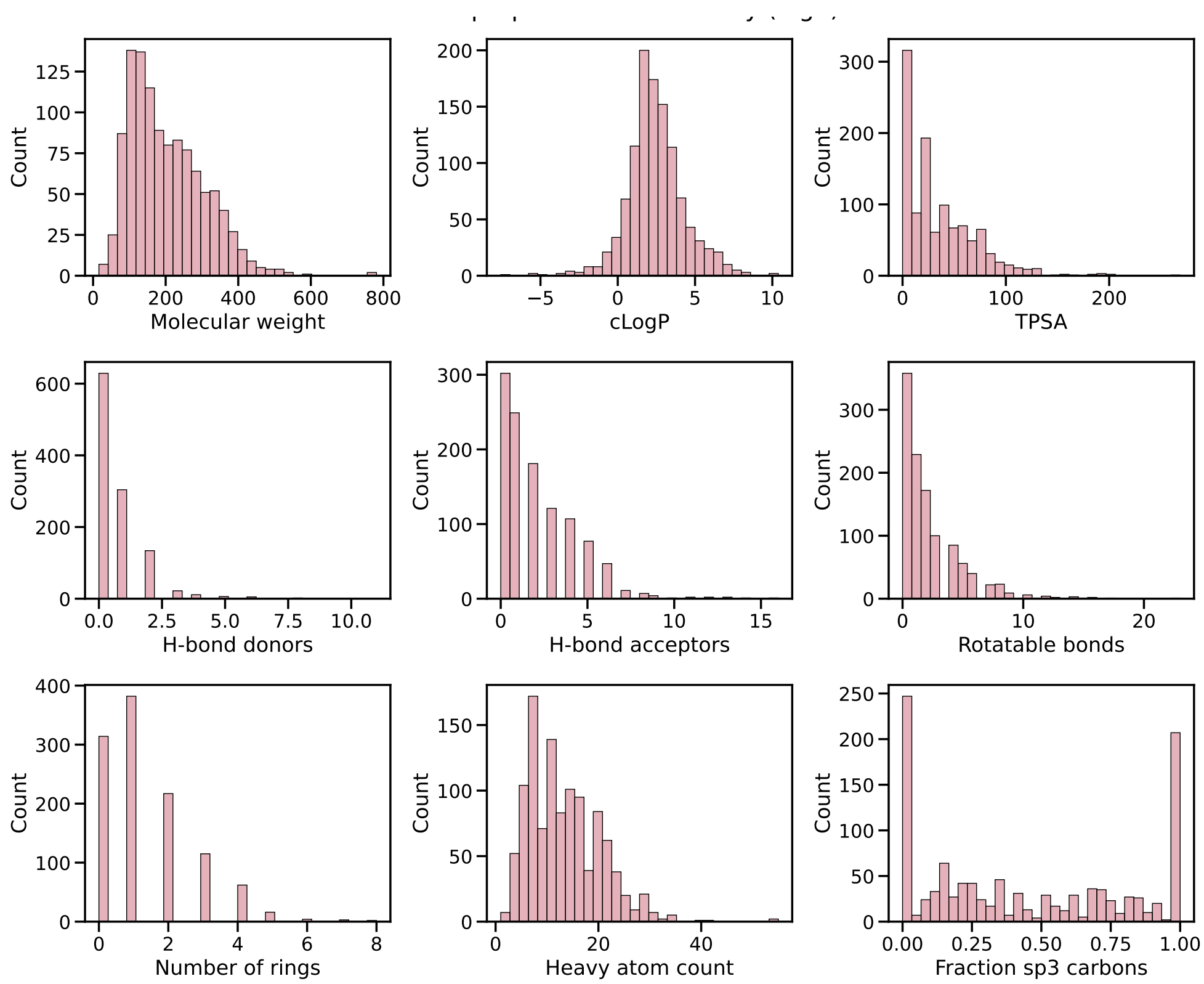

Figure S1: Distributions of physico-chemical properties for the aqueous solubility dataset.

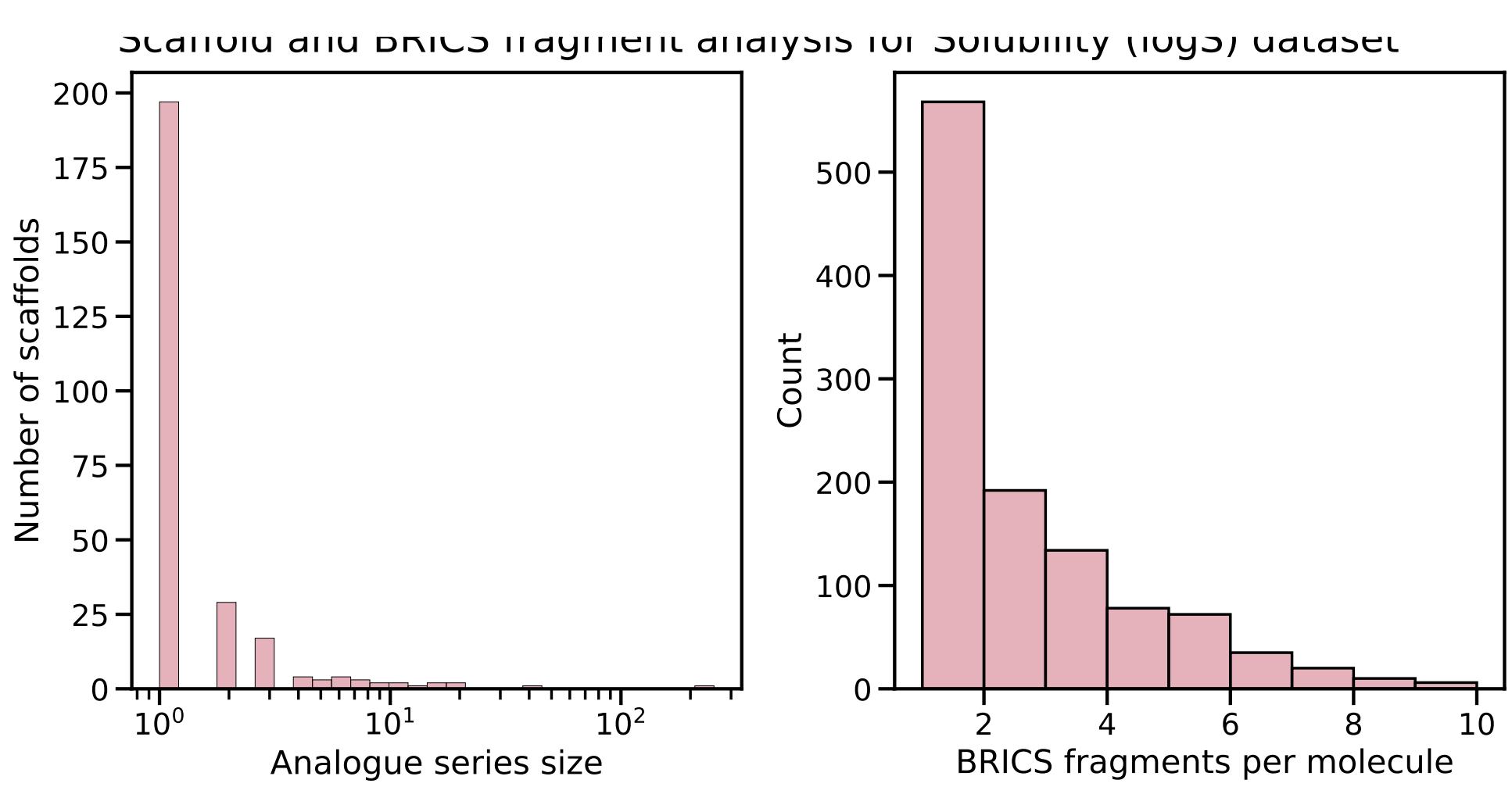

Figure S2: Distributions of Bemis–Murcko scaffolds and BRICS fragments for the aqueous solubility dataset.

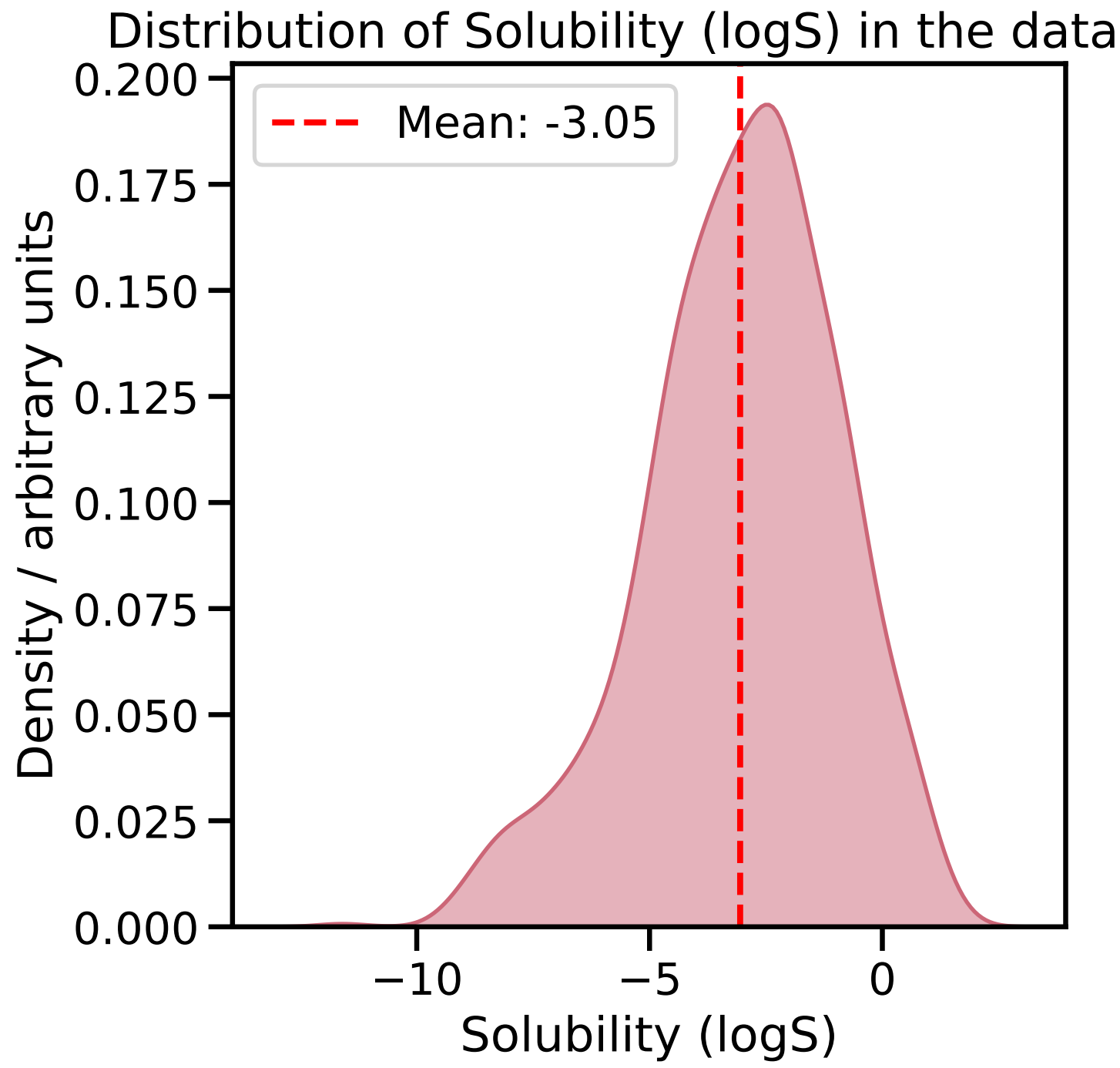

Figure S3: Distribution of the target value log(S) for the aqueous solubility dataset.

Mutagenicity

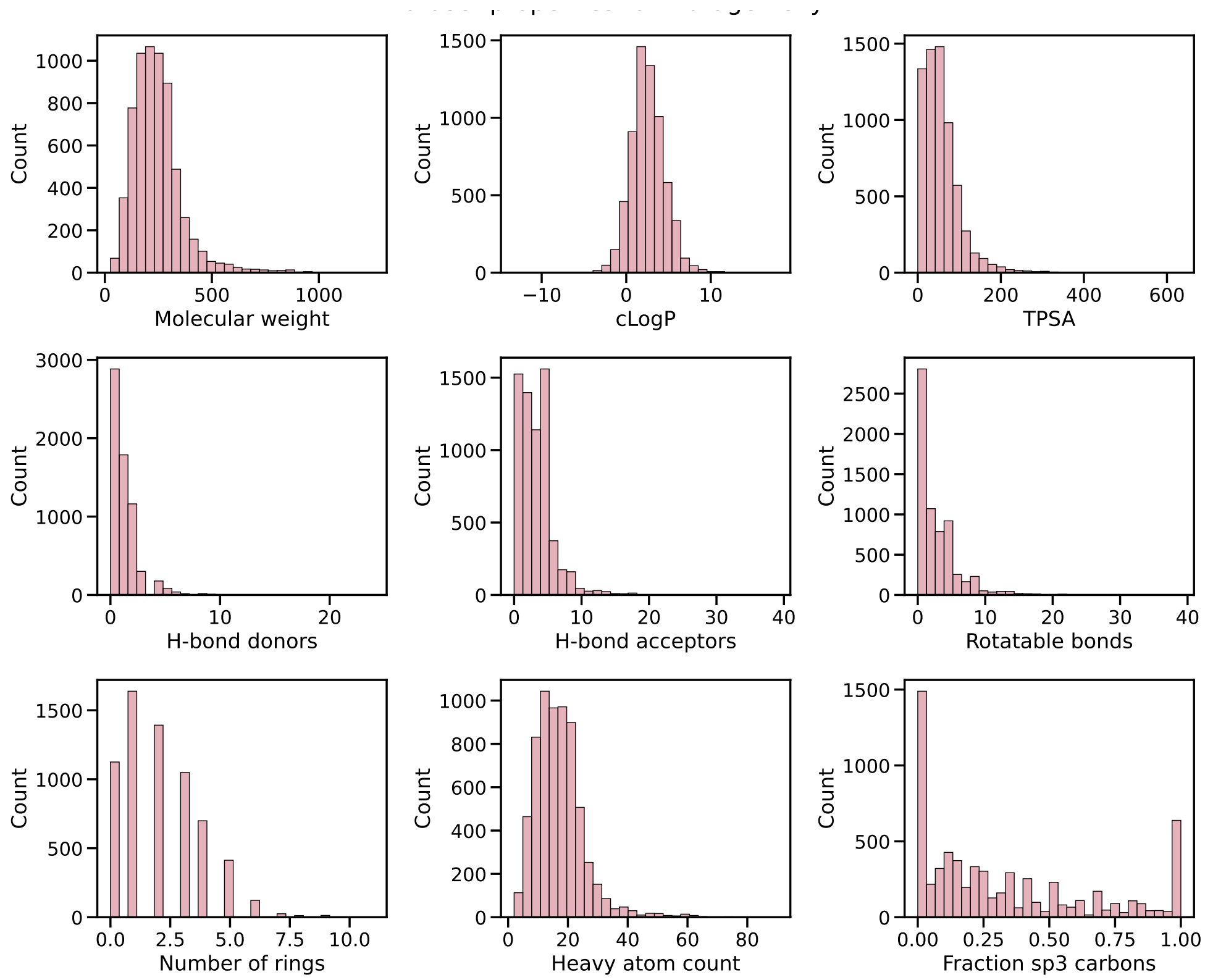

Figure S4: Distributions of physico-chemical properties for the mutagenicity dataset.

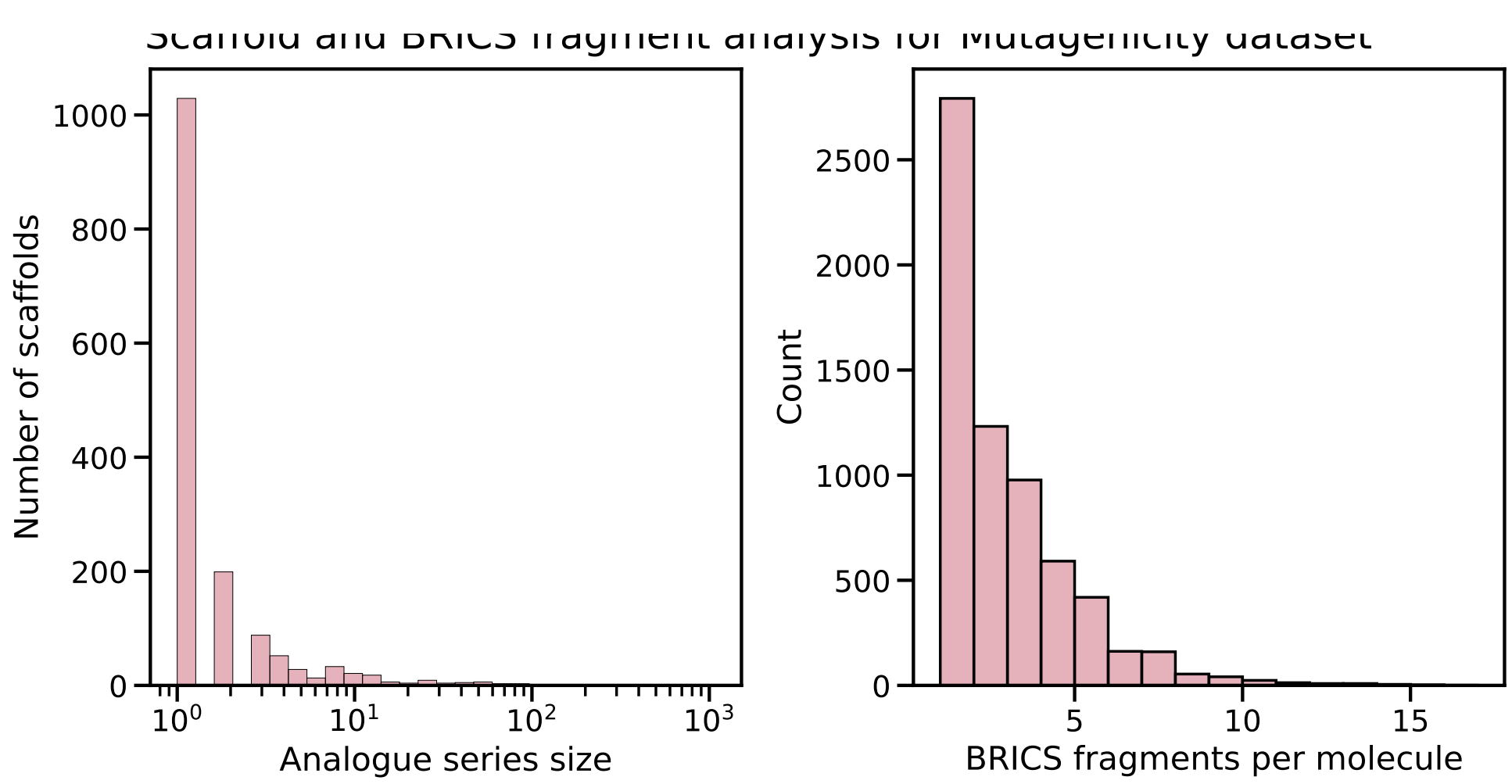

Figure S5: Distributions of Bemis–Murcko scaffolds and BRICS fragments for the mutagenicity dataset.

Antiviral Potency

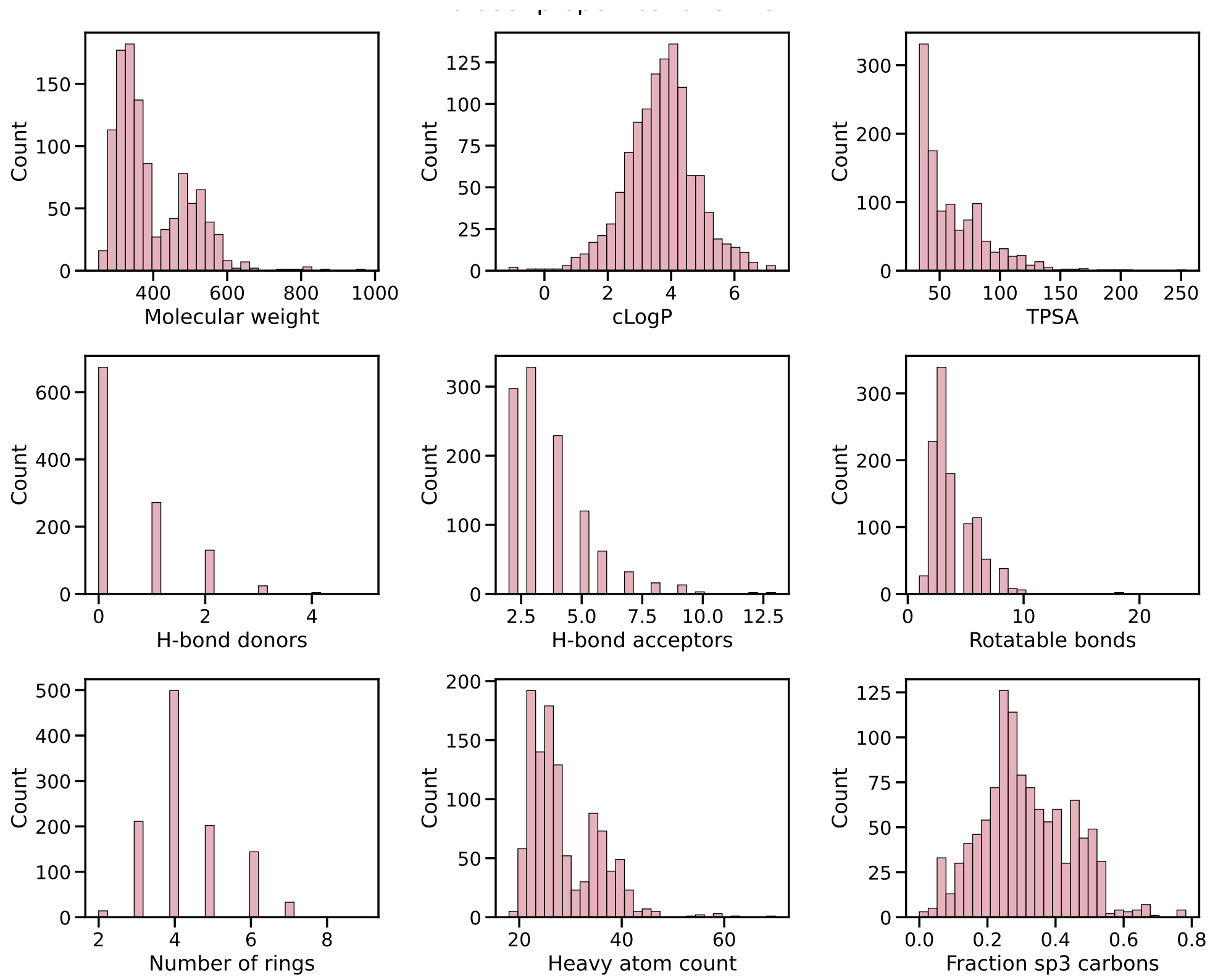

Figure S6: Distributions of physico-chemical properties for the antiviral potency dataset.

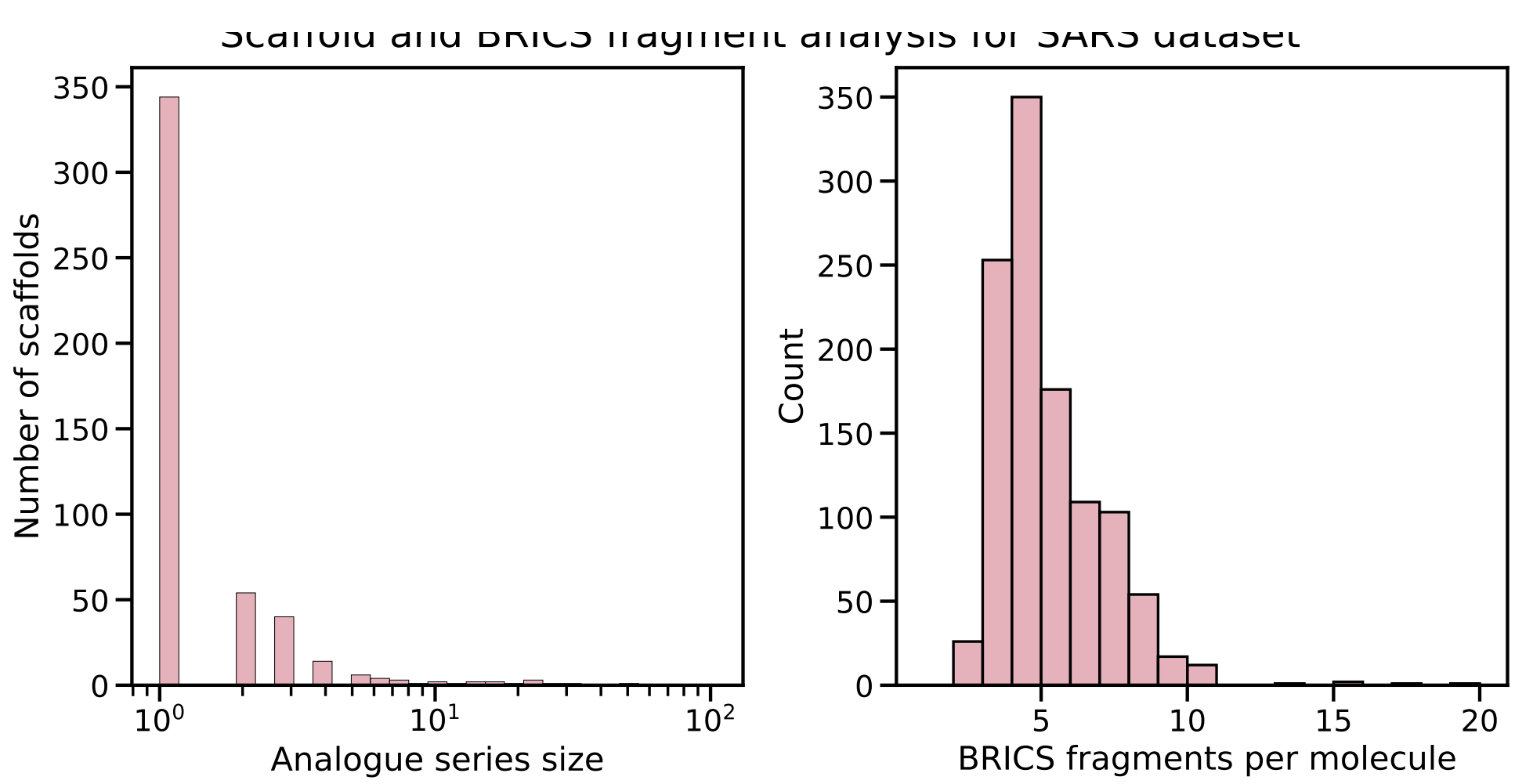

Figure S7: Distributions of Bemis–Murcko scaffolds and BRICS fragments for the antiviral potency dataset.

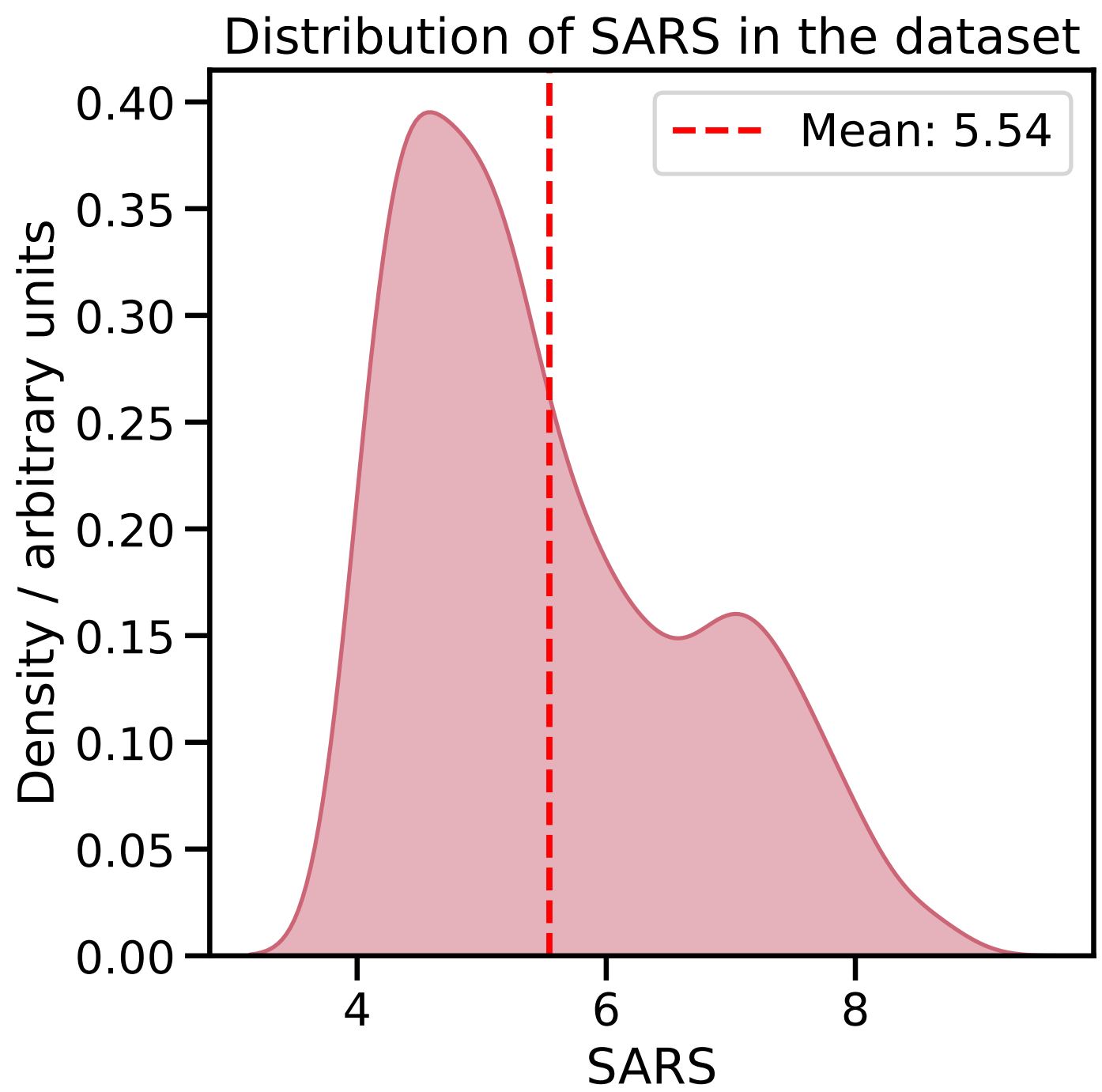

Figure S8: Distribution of experimental pIC50 values for the antiviral potency dataset.

Predictive Performance on Regression Tasks

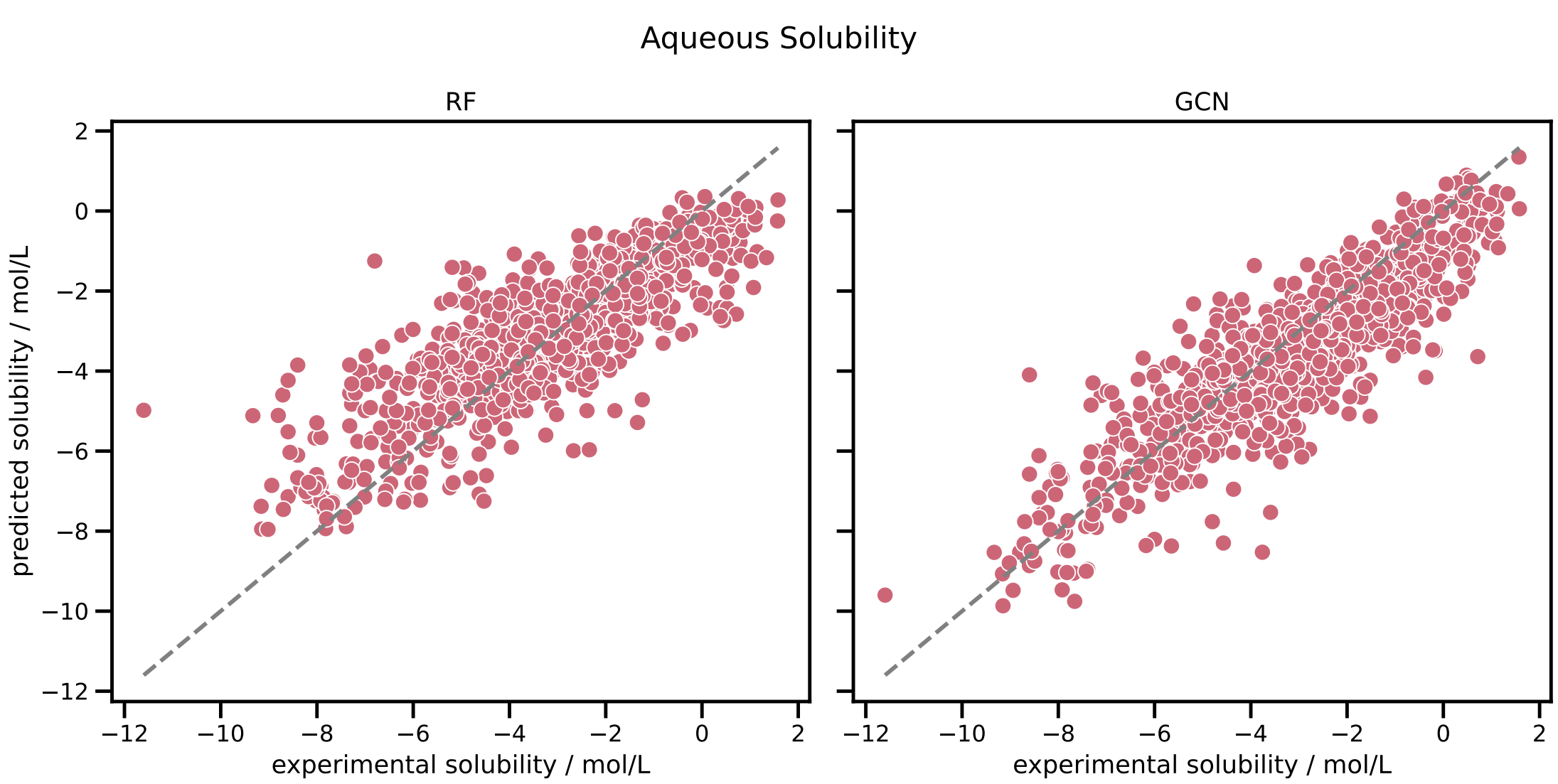

Figure S9: Scatter plot of predicted versus experimental aqueous solubility for the RF (left) and GCN (right) models.

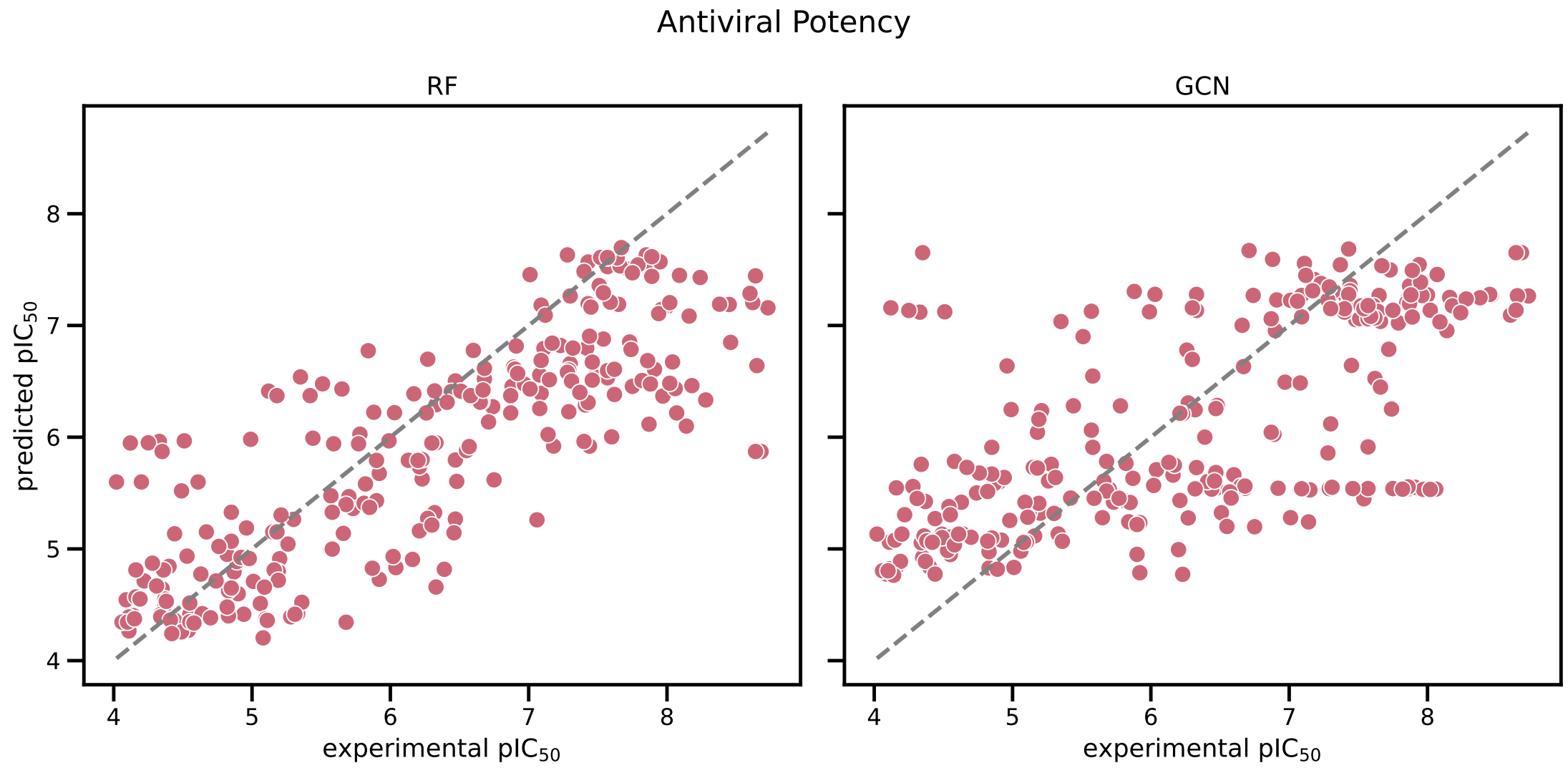

Figure S10: Scatter plot of predicted versus experimental pIC_{50} values for the RF (left) and GCN (right) models on the antiviral potency dataset.

Analysis of Solubility Trends across Analogue Series

To provide a more systematic evaluation of solubility trends within similar compounds, we performed an analogue-series analysis based on Bemis—Murcko scaffolds[2]. For each analogue series, the sum of the mean Shapley values of all non-scaffold fragments was compared with the corresponding model predictions.

Table S2: Quantitative assessment of the relationship between predicted aqueous solubility and the sum of mean non-scaffold fragment Shapley values for analogue series identified using Bemis–Murcko scaffolds.

model	scaffold	Pearson r	p-value	Kendall τ	p-value	n
RF	c1ccccc1	0.788	3.5×10^{-13}	0.557	9.8×10^{-10}	57
RF	O=C1CC(=O)NC(=O)N1	0.518	4.8×10^{-2}	0.429	2.7×10^{-2}	15
RF	c1ncncn1	0.357	3.5×10^{-1}	0.333	2.6×10^{-1}	9
GCN	c1ccccc1	0.871	1.2×10^{-18}	0.659	4.7×10^{-13}	57
GCN	O=C1CC(=O)NC(=O)N1	0.620	1.4×10^{-2}	0.810	2.3×10^{-6}	15
GCN	c1ncncn1	0.259	5.0×10^{-1}	0.278	3.6×10^{-1}	9

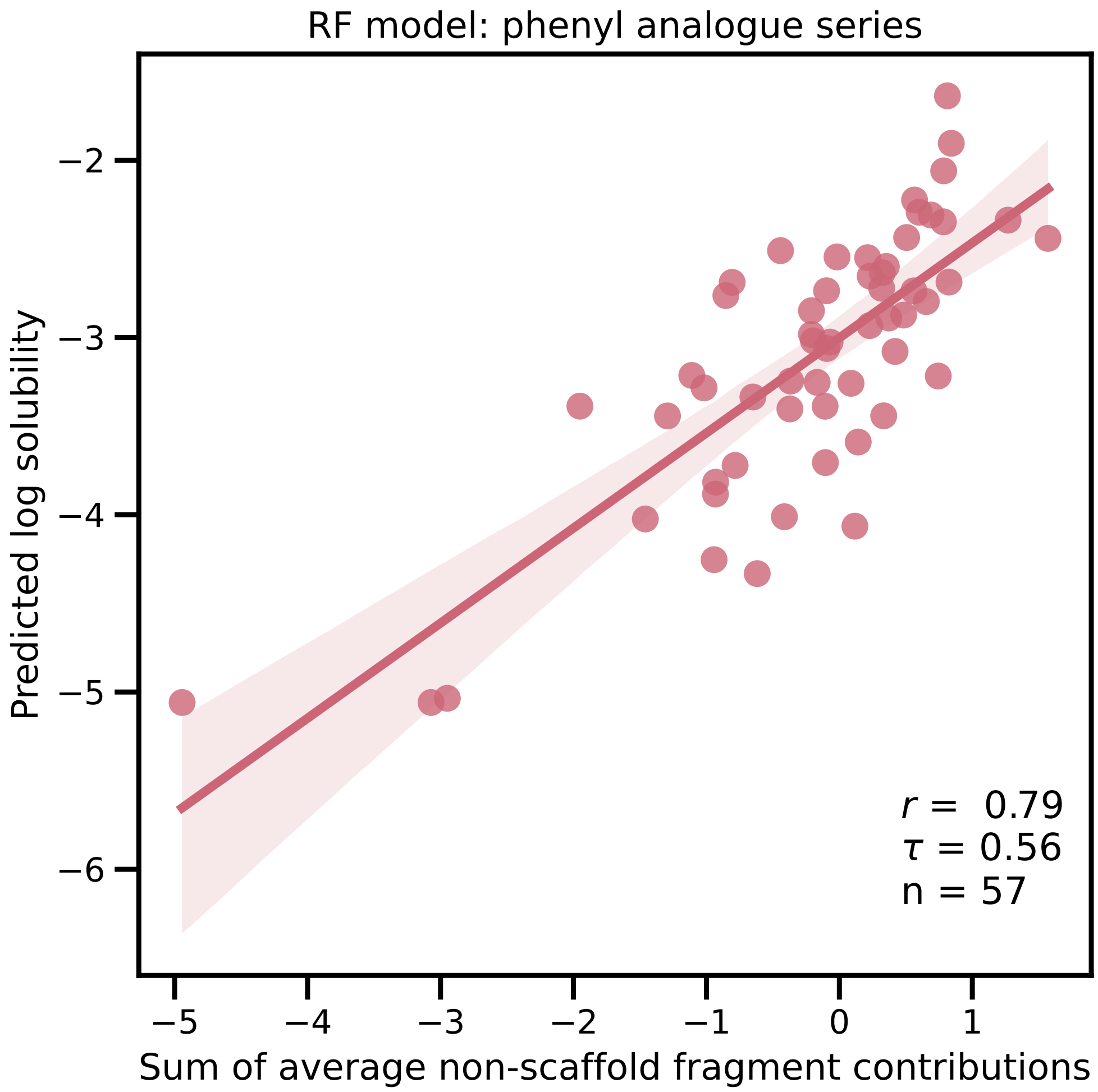

Figure S11: Relationship between predicted log solubility and the sum of average non-scaffold fragment Shapley values for compounds belonging to the phenyl analogue series (n = 57) on the RF model. Average fragment contributions were computed across all molecules in the dataset and summed for the substituents of each compound.

Sensitivity Analysis on the Empty Coalition Value

To evaluate the sensitivity of fragment-level Shapley values to the choice of empty-coalition value, we performed an additional analysis using an RF model trained on the aqueous solubility dataset. For this model, the prediction associated with an empty fingerprint was $\nu(\emptyset) = -1.06$. Fragment-level Shapley values were subsequently recomputed using an alternative empty-coalition value of $\nu(\emptyset) = 0.0$ and compared with the original attributions.

The resulting fragment-level Shapley values remained highly correlated between the two baseline definitions (Pearson $r = 0.98$, Kendall $\tau = 0.85$, Figure S13). Similar agreement was observed for mean fragment contributions across the dataset (Pearson $r = 0.99$, Kendall $\tau = 0.85$, Figure S12), indicating that the principal attribution trends are largely invariant to the choice of empty-coalition value.

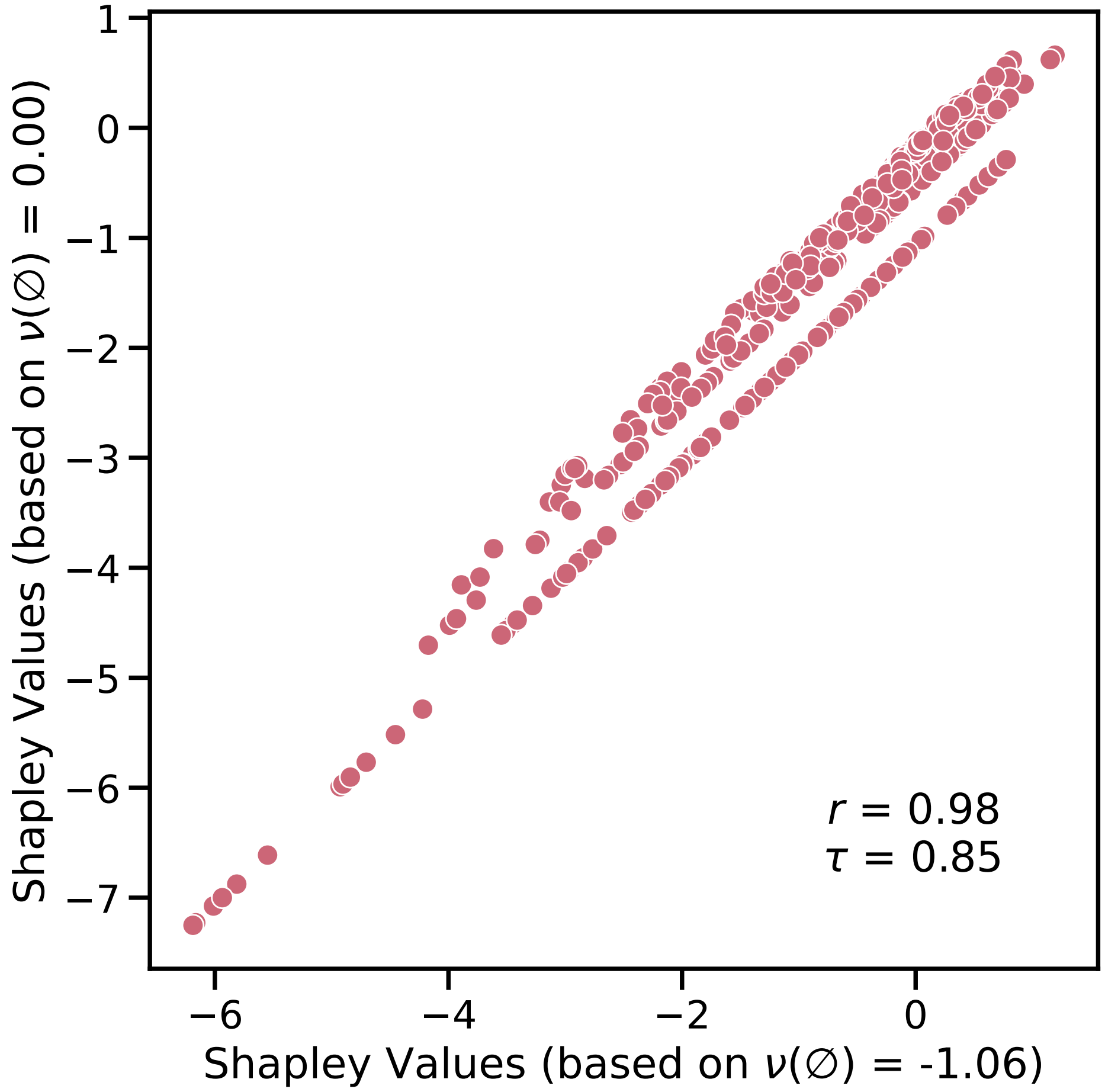

Figure S12: Comparison of fragment-level Shapley values obtained using the original and modified empty-coalition values. Each point corresponds to a fragment occurrence in the test set.

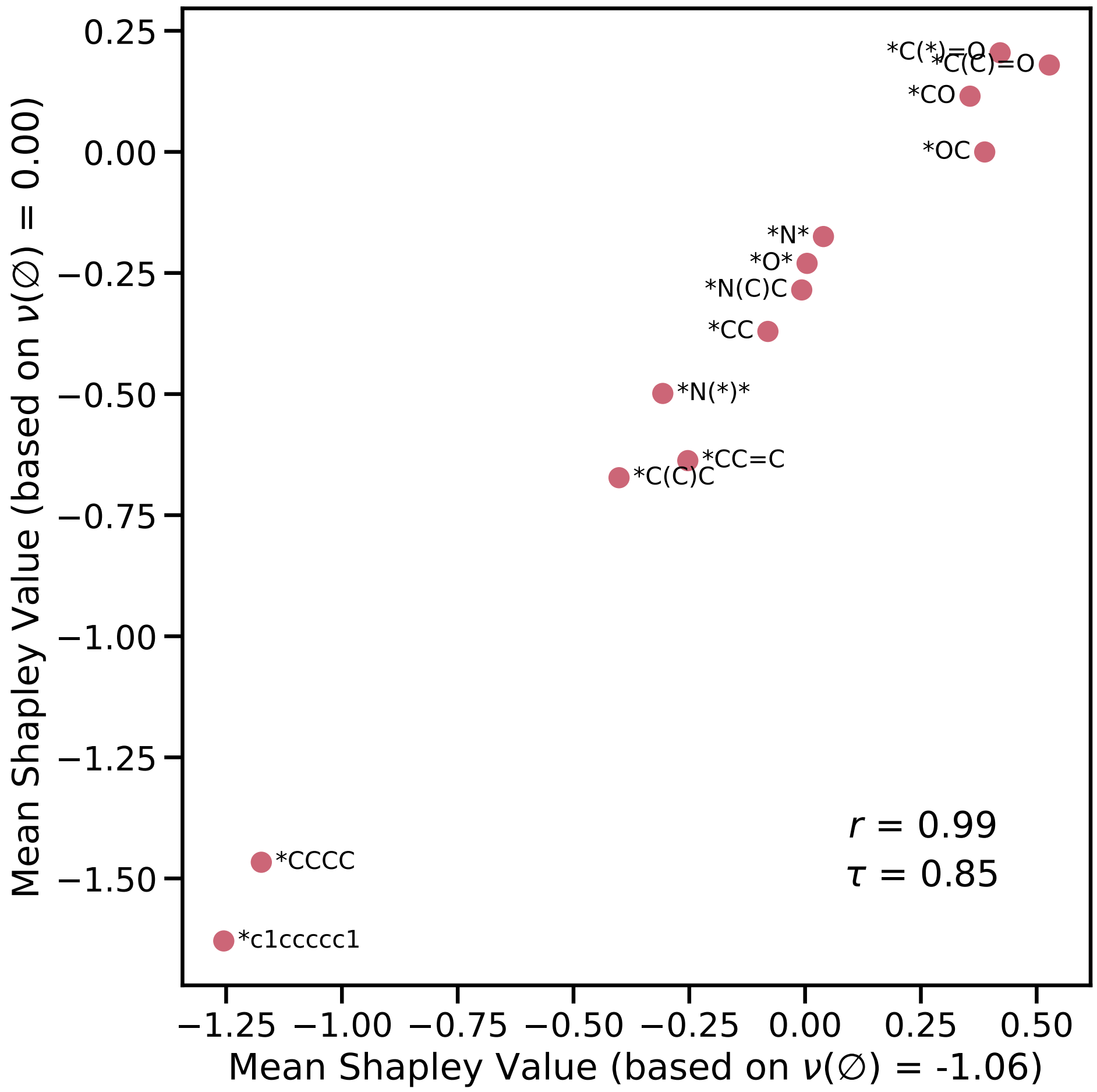

Figure S13: Comparison of mean fragment-level Shapley values for fragments appearing at least five times in the test set using different empty-coalition values.

dataset	model	mean $\nu(\emptyset)$	std $\nu(\emptyset)$
aqueous solubility	RF	-1.211	0.167
aqueous solubility	GCN	-0.939	0.482
mutagenicity	RF	0.081	0.024
mutagenicity	GCN	0.350	0.274
antiviral potency	RF	5.679	–
antiviral potency	GCN	5.024	–

Table S3: Mean and standard deviation of the empty coalition value $\nu(\emptyset)$.

Analysis of Reliance on Toxicophores for Mutagenicity Prediction

To assess the extent to which the models rely on established toxicophore motifs, we evaluated model performance separately for compounds containing and lacking at least one toxicophore reported by Kazius et al.[3]

Table S4: Distribution of toxicophores and mutagenic compounds in the mutagenicity dataset.

	ratio mutagenic	ratio non-mutagenic
entire dataset	53.8 %	46.2 %
$n_{\text{toxicophore}} = 0$	22.7 %	77.3 %
$n_{\text{toxicophore}} \geq 1$	75.7 %	24.3 %

Table S5: Performance metrics on the mutagenicity prediction task evaluated separately for compounds containing and lacking known toxicophore motifs.

model	metric	toxicophore containing	no toxicophore containing
RF	ROC-AUC	0.703 ± 0.015	0.712 ± 0.026
RF	Precision	0.862 ± 0.010	0.622 ± 0.048
RF	Recall	0.894 ± 0.024	0.527 ± 0.059
GCN	ROC-AUC	0.650 ± 0.015	0.668 ± 0.051
GCN	Precision	0.839 ± 0.008	0.495 ± 0.080
GCN	Recall	0.870 ± 0.017	0.494 ± 0.106

References

(1) Degen, J.; Wegscheid-Gerlach, C.; Zaliani, A.; Rarey, M. *ChemMedChem* **2008**, *3*, 1503–1507, DOI: 10.1002/cmdc.200800178.

(2) Bemis, G. W.; Murcko, M. A. *Journal of Medicinal Chemistry* **1996**, *39*, 2887–2893, DOI: 10.1021/jm9602928.

(3) Kazius, J.; McGuire, R.; Bursi, R. *Journal of Medicinal Chemistry* **2004**, *48*, 312–320, DOI: 10.1021/jm040835a.
